# The probability distribution of the ancestral population size conditioned on the reconstructed phylogenetic tree with occurrence data

**DOI:** 10.1016/j.jtbi.2020.110400

**Published:** 2021-01-21

**Authors:** Marc Manceau, Ankit Gupta, Timothy Vaughan, Tanja Stadler

**Affiliations:** Department of Biosystems Science and Engineering, ETH Zürich, Basel, Switzerland

**Keywords:** Birth-death process, Fossilized birth-death model, Epidemiology, Macroevolution, Phylogenetics

## Abstract

We consider a homogeneous birth-death process with three different sampling schemes. First, individuals can be sampled through time and included in a reconstructed phylogenetic tree. Second, they can be sampled through time and only recorded as a point ‘occurrence’ along a timeline. Third, extant individuals can be sampled and included in the reconstructed phylogenetic tree with a fixed probability. We further consider that sampled individuals can be removed or not from the process, upon sampling, with fixed probability. We derive the probability distribution of the population size at any time in the past conditional on the joint observation of a reconstructed phylogenetic tree and a record of occurrences not included in the tree. We also provide an algorithm to simulate ancestral population size trajectories given the observation of a reconstructed phylogenetic tree and occurrences. This distribution can be readily used to draw inferences about the ancestral population size in the field of epidemiology and macroevolution. In epidemiology, these results will allow data from epidemiological case count studies to be used in conjunction with molecular sequencing data (yielding reconstructed phylogenetic trees) to coherently estimate prevalence through time. In macroevolution, it will foster the joint examination of the fossil record and extant taxa to reconstruct past biodiversity.

## Highlights

• Reconstructed phylogenetic trees contain valuable information on population dynamics.
• Considering records of occurrences through time allows us to get more relevant data.
• Data is modeled using a birth-death process with specific sampling scheme.
• The distribution of the population size conditioned on the data is derived.
• This distribution will foster new advances in macroevolution and epidemiology.

## 1 Introduction

Owing to seminal papers by Yule, 1925, Kendall, 1948, and much later by Nee et al. (1994), birth-death models have become ubiquitous in evolutionary biology. They are used as a population dynamic model, parameterized via a birth and death rate, in studies spanning fields as diverse as paleontology, macroevolution, linguistics, and epidemiology (see e.g. Foote, 2000, Heath et al., 2014, Gray et al., 2009, Stadler et al., 2013). A major aim when using these models is to reliably estimate the ancestral number of species, languages or infected individuals, i.e. past biodiversity, past prevalence, or more general past population sizes. In both macroevolution and epidemiology, population dynamics inferences can rely on occurrence data, i.e. the fossil record and the case counts record. This data is modeled as a sampling of individuals from the full population through time (Foote, 2000, Starrfelt and Liow, 2016).

In recent years, impressive sequencing efforts targeting present-day species and pathogens have enabled the reconstruction of phylogenies. Two main modeling approaches allow to quantify past population sizes in the past using these trees. First, phylodynamic tools have been developed to fit the birth and death rates of a birth-death process on the reconstructed phylogenetic tree of interest, while integrating over past population sizes (Stadler, 2011, Morlon et al., 2011). In order to quantify past population sizes, typically the expected population sizes based on these estimated birth and death rates are calculated (Morlon et al., 2011, Ratmann et al., 2016, Billaud et al., 2019).

Thus, such population sizes are not directly conditioned on the reconstructed phylogenetic tree. Instead, the statistical signal in the tree is only used to compute rate estimates. Second, phylodynamic tools have been developed to fit the expected population size of a coalescent model on a reconstructed phylogenetic tree. This modeling approach may appear as a better alternative, for it is directly parametrized with the population size that we wish to estimate. However, this comes at the cost of ignoring stochastic fluctuations in small populations (Morlon et al., 2010, Ratmann et al., 2016).

Statistical approaches stemming from the analysis of case count data or from the analysis of reconstructed evolutionary trees have been part of separate bodies of work for many years, historically yielding conflicts between biodiversity estimates based on the fossil record and estimates based on reconstructed phylogenies of extant taxa (Quental and Marshall, 2010 but see also Morlon et al., 2011). A first path towards merging these disparate data was introduced by the fossilized birth-death model of Stadler (2010), which considered a birth-death model with sampling and inclusion of individuals in the tree through time. This allowed taking into account infection trees reconstructed from pathogen sequences sampled throughout an epidemic (Stadler et al., 2011). In macroevolution, it paved the way to more precise phylogenetic dating using well-conserved fossil taxa which could be placed on a reconstructed phylogeny using morphological characters (Gavryushkina et al., 2016). Not so well-conserved fossils (i.e. occurrences) have also been used with this model, using a Markov Chain Monte Carlo (MCMC) scheme to integrate over all possible placements along a fixed tree (Heath et al., 2014). Analytical developments around this new model have been made by Gupta et al. (2019), which derived an analytical formula for the probability density of an outcome of the process, which consists of a reconstructed phylogenetic tree along with a record of occurrences. Again, all these methods do not quantify population sizes directly, but estimate birth and death rates while analytically integrating over population sizes.

Very recently, Vaughan et al. (2019) introduced a Monte-Carlo particle filtering algorithm allowing direct quantification of past population sizes and birth and death rates conditioned on reconstructed phylogenetic trees and occurrences (see Andrieu et al., 2010 for details about particle filtering methods). As such, it can produce more accurate population size estimates than the methods mentioned above as the estimates directly condition on all data, i.e. the occurrence record (e.g. poorly preserved fossils, or case count epidemiological record) and the reconstructed phylogenetic tree.

In this paper, we build on the analytical developments presented by Gupta et al. (2019), to calculate the past population size distribution as originally targeted by Vaughan et al. (2019). Our approach here is more analytic, leading to much faster numerical calculations compared to the particle filtering method previously developed. The efficiency of our method paves the way towards considering much bigger datasets, and towards extending the method to multi-type or density-dependent birth-death processes.

In Section 2, we present the model, notation, and an overview of the strategy to express the targeted distribution. In Section 3, we adapt the main results of Gupta et al. (2019) to compute the probability density of observations made after a given time, conditioned on the past population size. In Section 4, we provide a way to compute the joint density of the past population size and observations made before a given time. Combining results of Sections 3, 4 in Section 5, we compute the distribution of past population sizes conditional on the full outcome of the process, and perform sanity checks against previously published methods achieving similar tasks (Stadler, 2010, Vaughan et al., 2019, Gupta et al., 2019). We finally discuss applications and potential extensions of the model.

## 2 Model and notation

### 2.1 Parameters of the process

We consider a population of individuals, any of which can give birth to another individual at rate λ or die at rate μ. The process starts at time $t_{\mathrm{or}}$ in the past with one individual, and evolves until reaching present time 0, i.e. time is oriented from the present towards the past. In the rest of the manuscript, something *happening at time t* will thus always refer to an event taking place *t units before present*.

We superimpose to this background population dynamics three different sampling schemes. First, individuals can be ψ*-sampled* at rate ψ throughout their lifetime. When ψ-sampled, the individual will be included in the reconstructed phylogenetic tree. Second, individuals can be ω*-sampled* at rate ω throughout their lifetime. When ω-sampled, the individual is not included in the reconstructed phylogenetic tree, but its sampling time is nevertheless recorded and called ‘an occurrence’. Last, the process finishes upon reaching the present time 0, and each extant individual at that time is ρ*-sampled* with fixed probability ρ, leading to their inclusion in the reconstructed phylogenetic tree. The sum of all per-capita rates will be called for short γ=λ+μ+ψ+ω.

Following Vaughan et al. (2019), we also include in the model an effect of the ψ- and ω-sampling through time on the population dynamics. We consider that, upon sampling, an individual is either removed from the process with probability r∈(0,1), or is unaffected by the sampling with probability (1-r). The overall number of individuals, denoted $\left(I_{t}\right)$, thus follows a linear birth-death process with birth rate λ and death rate μ+(ψ+ω)r. Note that, because the ρ-sampling step occurs here at the end of the process, it does not matter whether or not individuals are removed upon ρ-sampling.

### 2.2 Introducing useful probabilities

Some aspects of this process have been previously investigated thoroughly. We now use two key probabilities. First, we will call $u_{t}$ the probability that a process starting at time *t* with only one individual remains unsampled up to and including the present time (time 0). We recall that $u_{t}$ satisfies the ordinary differential equation (ODE) (Maddison et al., 2007)

(2.1) $$\begin{array}{cc} & u_{0}=z\\ & {\overset{˙}{u}}_{t}=\lambda u_{t}^{2}-\gamma u_{t}+\mu \text{.} \end{array}$$

The solution of this for a particular initial condition *z* being the following

(2.2) $$u(t,z)=\frac{x_{1}(x_{2}-z)-x_{2}(x_{1}-z)e^{-\sqrt{\Delta }t}}{(x_{2}-z)-(x_{1}-z)e^{-\sqrt{\Delta }t}}$$

where $\Delta =\gamma^{2}-4\lambda \mu >{\left(\lambda +\mu \right)}^{2}-4\lambda \mu ⩾{\left(\lambda -\mu \right)}^{2}>0$ and $x_{1},x_{2}$ are the two roots of the polynomial $\lambda x^{2}-\gamma x+\mu$,

$$x_{1}=\frac{\gamma -\sqrt{\Delta }}{2\lambda }\;\mathrm{and}\;x_{2}=\frac{\gamma +\sqrt{\Delta }}{2\lambda }\text{.}$$

Second, we call $p_{t}$ the probability that a process starting at time *t* with one individual precisely leads to one sampled individual at present time 0. Writing the ODE governing the evolution of this quantity leads to

(2.3) $$\begin{array}{cc} & p_{0}=1-z\\ & {\overset{˙}{p}}_{t}=(2\lambda u(t,z)-\gamma )p_{t}\text{.} \end{array}$$

The solution of this being the following

(2.4) $$p(t,z)=(1-z)\frac{\Delta }{\lambda^{2}}{\left((x_{2}-z)-(x_{1}-z)e^{-\sqrt{\Delta }t}\right)}^{-2}e^{-\sqrt{\Delta }t}\text{.}$$

These formulas are well known, and correspond respectively to quantities called $p_{0}(t)$ and $p_{1}(t)$ in Stadler (2010). When z=1-ρ, we will drop the dependence on *z* and use the shorter notation $u_{t},p_{t}$. We recall standard ways to derive these expressions in Appendix A.

### 2.3 Strategy of the paper

**Fig. 1:**
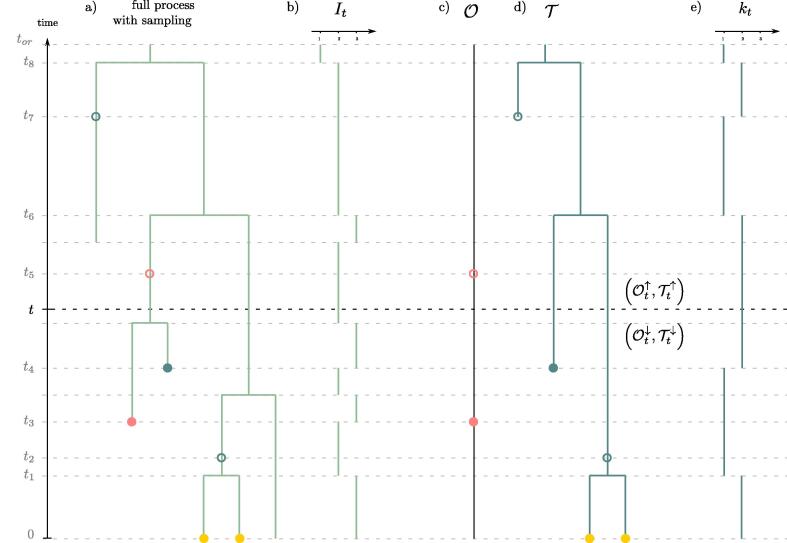
General setting of the method. a) the full process with sampling. Pink dots translate as dots in O and correspond to ω-sampling (sampling through time without sequencing). Blue dots translate as dots in T and correspond to ψ-sampling (sampling through time with sequencing). Yellow dots correspond to all present-day ρ-sampling events. Filled or unfilled dots correspond respectively to sampling with or without removal. b) Population size through time. c) Observed occurrences through time. d) Reconstructed phylogenetic tree. e) Number of individuals in reconstructed phylogenetic tree through time. (For interpretation of the references to colour in this figure legend, the reader is referred to the web version of this article.)

The process with sampling leads to the observation of two distinct objects (T,O) illustrated in Fig. 1.

The reconstructed phylogenetic tree T, on the one hand, represents the evolutionary relationships between all ψ-sampled and ρ-sampled individuals. We further consider that ψ-sampled individuals are labeled either as ‘removed’ or ‘non-removed’. All ψ-sampled removed individuals are necessarily leaves of T, whereas ψ-sampled non-removed ones can either stand as leaves (when the descent of the individual is not sampled) or as vertices along a branch (when the descent of the individual is further sampled), in which case they are referred to as *sampled ancestors*.

The record of occurrences O, on the other hand, is an ordered list of all ω-sampling times. We also consider that these sampling times are labeled as either ‘removed’ or ‘non-removed’.

In this paper, we are interested in computing the probability distribution of the number of individuals in the past, conditioned on the observed outcome (T,O) of the process. If $k_{t}$ denotes the number of sampled lineages in T at time *t*, we call our target distribution,

(2.5) $$\forall t⩾0,\forall i\in N_{0}=\{0,1,2,\ldots \},\;K_{t}^{\left(i\right)}≔P(I_{t}=k_{t}+i|T,O)\text{.}$$

We will refer to *epochs* as the maximal time slices within which no sampling event in O, nor branching event in T, happened. These epochs are delimited by the union of sampling times in O, branching times in the tree T, and sampling times of leaves and sampled ancestors in T. All pooled together, we call these ordered times ${\left(t_{h}\right)}_{h=0}^{n}$, starting at present time $t_{0}=0$ and ending at the origin time $t_{n}=t_{\mathrm{or}}$.

At any time t⩾0 we also introduce:

$$\begin{array}{cc} & T_{t}^{↑}≔\text{the tree}\;T\;\text{starting at the origin time}\;t_{\mathrm{or}}\;\text{and cut at time}\;t\\ & T_{t}^{↓}≔\text{the collection of trees}\;(\text{or forest})\;\text{obtained by cutting}\;T\\ & \;\;\text{at time}\;t,\;\text{and considering all subtrees descending from cut lineages}\\ & O_{t}^{↑}≔O_{\left|(t,t_{\mathrm{or}}\right)}\\ & O_{t}^{↓}≔O_{\left|(0,t\right)} \end{array}$$

The general strategy – and outline – of the paper is the following. We will traverse the tree and record of occurrences *breadth-first*, i.e. level-by-level through time. In a *backward traversal* we will compute the probability density of observations made between time *t* and 0 conditioned on the population size at time *t*. We call this probability density,

(2.6) $$\forall i\in N_{0},\;L_{t}^{\left(i\right)}≔P\left(T_{t}^{↓},O_{t}^{↓}|I_{t}=k_{t}+i\right)\text{.}$$

In a *forward traversal* we will then compute the joint probability density of the observations made prior to time *t* and the population size at time *t*. We call this density,

(2.7) $$\forall i\in N_{0},\;M_{t}^{\left(i\right)}≔P\left(T_{t}^{↑},O_{t}^{↑},I_{t}=k_{t}+i\right)\text{.}$$

Provided we get expressions of ${\left(L_{t}\right)}_{t=0}^{t_{\mathrm{or}}}$ and ${\left(M_{t}\right)}_{t=0}^{t_{\mathrm{or}}}$, our target distribution can then be expressed by combining both, noting that

(2.8) $$\begin{array}{cc} K_{t}^{\left(i\right)}≔ & P\left(I_{t}=k_{t}+i|T,O\right)\\ & \propto P\left(I_{t}=k_{t}+i,T_{t}^{↑},O_{t}^{↑},T_{t}^{↓},O_{t}^{↓}\right)\\ & =P\left(T_{t}^{↓},O_{t}^{↓}|I_{t}=k_{t}+i,\;T_{t}^{↑},O_{t}^{↑}\right)P\left(I_{t}=k_{t}+i,\;T_{t}^{↑},O_{t}^{↑}\right)\\ & =L_{t}^{\left(i\right)}M_{t}^{\left(i\right)} \end{array}$$

where the last line holds because, conditionally on $I_{t}=k_{t}+i$, the future of the (Markov) process is independent of what happened before.

In the process of getting the probability density of T,O under the same model, Gupta et al. (2019) provided an analytical formula and an algorithm to compute the first ingredient $L_{t}$ in the case where all individuals are removed upon sampling (i.e. r=1). We thus recall their main result, and adapt it to our slightly different framework, in the next section.

## 3 Calculation of $L_{t}$ – The density of observations below *t* conditioned on past population size

We start this section by presenting the ODEs satisfied by the probability density $L_{t}$. This provides us with a numerical algorithm to compute $L_{t}$, which we subsequently simplify with analytical results for specific sets of parameters.

### 3.1 Set of ODEs satisfied by $L_{t}$

We can derive the probability density $L_{t}$ by studying its evolution through time. First, observe that we can express $L_{0}$ at present time 0. Indeed, provided we know the exact number of individuals living at time 0, the probability to see the tips of the tree is directly driven by the ρ-sampling,

(3.1) $$\forall i\in N_{0},\;L_{0}^{\left(i\right)}=\rho^{k_{0}}{\left(1-\rho \right)}^{i}\text{.}$$

**Fig. 2:**
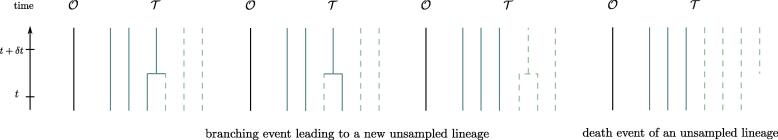
Four unobservable scenarios taken into account to derive the ODEs (3.2), (4.1).

We now derive the ODE driving the evolution of $L_{t}$ through time across any given epoch. We consider an infinitesimal time step δt and list the events which could have happened in the full process between t+δt and *t*, leading to our observations. Suppose the number of observed lineages in this epoch is *k*, and the total number of individuals alive is k+i. We emphasize three cases, illustrated in Fig. 2:

1. nothing happened with probability $\left(1-\gamma (k+i)\delta t\right)$
2. a birth event happened
  (a) among the *k* sampled lineages in $T_{t}^{↓}$, and it leads to an extinct or unsampled subtree to the left or to the right, with probability 2λkδt.
  (b) among the *i* other individuals, with probability λiδt.
3. a death event happened among the *i* particles, with probability μiδt.

These allow us to write, $\forall i\in N_{0}$,

$$L_{t+\delta t}^{\left(i\right)}=\left(1-\gamma (k+i)\delta t\right)L_{t}^{\left(i\right)}+\lambda (2k+i)\delta {\mathrm{tL}}_{t}^{\left(i+1\right)}+\mu i\delta {\mathrm{tL}}_{t}^{\left(i-1\right)}\text{.}$$

Note that for $i=0,\;L_{t}^{\left(i-1\right)}$ is not defined, but the term cancels out thanks to the factor *i*.

Subtracting $L_{t}^{\left(i\right)}$ from both sides, dividing by δt and letting δt→0, we get the following set of ODEs driving the evolution of $L_{t}$,

(3.2) $$\begin{array}{cc} \forall i\in N_{0},\;L_{0}^{\left(i\right)}= & \rho^{k_{0}}{\left(1-\rho \right)}^{i}\\ {\overset{˙}{L}}_{t}^{\left(i\right)}= & -\gamma (k+i)L_{t}^{\left(i\right)}+\lambda (2k+i)L_{t}^{\left(i+1\right)}+\mu {\mathrm{iL}}_{t}^{\left(i-1\right)}\text{.} \end{array}$$

Last, we need to study how $L_{t}$ changes at punctual events. We call *unsampled lineages* the lineages that do not appear on the reconstructed phylogenetic tree, i.e. have not been ρ- or ψ-sampled. Note that these unsampled lineages might still be subject to ω-sampling events.

**Fig. 3:**
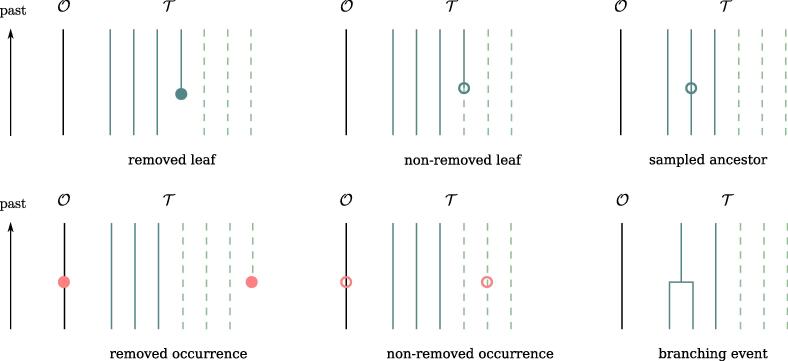
Six observable punctual events in the data.

There are 6 types of punctual events that we can come across at time *t* in the past, listed below and illustrated in Fig. 3. We denote $L_{t^{+}}$ the probability just before (i.e. up) the punctual event and $L_{t^{-}}$ the probability immediately after (i.e. down). One directly gets $L_{t^{+}}$ by decomposing it into what must occur below $t^{-}$, multiplied by the rate of the specific event happening on the infinitesimal time window $\left(t^{-},t^{+}\right)$. We can either find,

1. a leaf of $T_{t}^{↓}$, labeled as removed. This is a ψ-sampling with removal event for which the number of unsampled lineages remains constant, and the number of sampled lineages increases by one (going backward in time). It thus gives,
  (3.3) $$L_{t^{+}}^{\left(i\right)}=\psi {\mathrm{rL}}_{t^{-}}^{\left(i\right)}\text{.}$$
2. a leaf of $T_{t}^{↓}$, labeled as non-removed. This is a ψ-sampling without removal event for which one of the unsampled lineage becomes a sampled one (going backward in time). It thus gives,
  (3.4) $$L_{t^{+}}^{\left(i\right)}=\psi (1-r)L_{t^{-}}^{\left(i+1\right)}\text{.}$$
3. a sampled ancestor along a branch of $T_{t}^{↓}$, necessarily labeled as non-removed. This is a ψ-sampling without removal event, not impacting the number of sampled or unsampled lineages. It thus gives,
  (3.5) $$L_{t^{+}}^{\left(i\right)}=\psi (1-r)L_{t^{-}}^{\left(i\right)}\text{.}$$
4. an occurrence in $O_{t}^{↓}$, labeled as removed. This is a ω-sampling with removal event, for which the number of unsampled lineages increases by one (going backward in time). It thus gives,
  (3.6) $$L_{t^{+}}^{\left(i\right)}=\omega {\mathrm{riL}}_{t^{-}}^{\left(i-1\right)}\text{.}$$
  Note that here also, for $i=0,L_{t}^{\left(-1\right)}$ is not defined but the term cancels out thanks to the factor *i*.
5. an occurrence in $O_{t}^{↓}$, labeled as non-removed. This is a ω-sampling without removal event, not impacting the number of sampled or unsampled lineages. It thus gives,
  (3.7) $$L_{t^{+}}^{\left(i\right)}=\omega (k+i)(1-r)L_{t^{-}}^{\left(i\right)}\text{.}$$
6. a branching event between two branches of $T_{t}^{↓}$. The number of sampled lineages decreases by one (going backward in time). It thus gives,
  (3.8) $$L_{t^{+}}^{\left(i\right)}=\lambda L_{t^{-}}^{\left(i\right)}\text{.}$$

Note that these updates can be adapted to the case when we don’t observe the removal status of individuals. The update corresponding to a leaf of T is the sum of updates (3.3), (3.4), the update corresponding to an occurrence event is the the sum of updates (3.6), (3.7), while updates (3.5), (3.8) are unchanged.

This set of ODEs (3.2) together with update Eqs. (3.3), (3.4), (3.5), (3.6), (3.7), (3.8) can be numerically approximated. To do so, we fix a finite upper bound *N* on the number of hidden individuals and numerically integrate a truncated ODE system. We detail this in the following algorithm to compute an approximation of $L_{t}$ at any time *t*.

**Table:** 

**Algorithm 1:** Computes a numerical approximation of $L_{t}$ for a specific set of times
**Input:**
Observed tree and occurrence data (T,O),
parameters $\left(t_{\mathrm{or}},\lambda ,\mu ,\psi ,\omega ,\rho ,r\right)$,
set of time points ${\left(\tau_{j}\right)}_{j=1}^{S}$ for which we want to compute the density $L_{\tau_{j}}^{\left(i\right)}$,
and the truncation *N* setting the accuracy of the algorithm.
**Output**: A numerical approximation of $L_{t}$ at times ${\left(\tau_{j}\right)}_{j=1}^{S},{\left({\overset{∼}{L}}_{\tau_{j}}^{\left(i\right)}\right)}_{\begin{array}{c} i\in \{0,1,\ldots ,N\}\\ j\in \{1,2,\ldots ,S\} \end{array}}$. 1: Pool all $\left(\tau_{j}\right)$ and all branching and sampling times of (T,O) in an ordered list ${\left(t_{h}\right)}_{h=1}^{n}$
2: Set j=1 and initialize *B* as a S×(N+1) empty matrix
3: Set $\forall i\in \{0,1,\ldots ,N\},\;{\overset{∼}{L}}_{0}^{\left(i\right)}=\rho^{k_{0}}{\left(1-\rho \right)}^{i}$
4: **for**h=1,2,…,n
5: Numerically solve the ODE ${\overset{˙}{\overset{∼}{L}}}_{t}=A{\overset{∼}{L}}_{t}$ on $\left(t_{h-1},t_{h}\right)$, by computing ${\overset{∼}{L}}_{t_{h}}=e^{(t_{h}-t_{h-1})A}{\overset{∼}{L}}_{t_{h-1}}$,
6: where matrix *A* is a (N+1)×(N+1) tridiagonal matrix with entries given by,
$$\begin{array}{cc} & \forall i\in \{0,1,\ldots ,N\}A^{\left(i,i\right)}=-\gamma (k+i)\\ & \forall i\in \{0,1,\ldots ,N-1\}A^{\left(i,i+1\right)}=\lambda (2k+i)\\ & \forall i\in \{1,2,\ldots ,N\}A^{\left(i,i-1\right)}=\mu i \end{array}$$
7: **if**$t_{h}=\tau_{j}$
8: Record $\forall i,B^{\left(j,i\right)}={\overset{∼}{L}}_{t_{h}}^{\left(i\right)}$
9: Set j=j+1
10: **end if**
11: **if**$t_{h}=t_{n}$ or $t_{h}=\tau_{S}$**then**
12: **return***B*
13: **else if**$t_{h}$ is a removed leaf **then**
14: Set ${\overset{∼}{L}}_{t_{h}^{+}}=\psi r{\overset{∼}{L}}_{t_{h}^{-}}$
15: **else if**$t_{h}$ is a non-removed leaf **then**
16: Set $\forall i<N,\;{\overset{∼}{L}}_{t_{h}^{+}}^{\left(i\right)}=\psi (1-r){\overset{∼}{L}}_{t_{h}^{-}}^{\left(i+1\right)}$ and ${\overset{∼}{L}}_{t_{h}^{+}}^{\left(N\right)}=0$
17: **else if**$t_{h}$ is a sampled ancestor **then**
18: Set ${\overset{∼}{L}}_{t_{h}^{+}}=\psi (1-r){\overset{∼}{L}}_{t_{h}^{-}}$
19: **else if**$t_{h}$ is a removed occurrence **then**
20: Set $\forall i>0,\;{\overset{∼}{L}}_{t_{h}^{+}}^{\left(i\right)}=\omega \mathrm{ri}{\overset{∼}{L}}_{t_{h}^{-}}^{\left(i-1\right)}$ and ${\overset{∼}{L}}_{t_{h}^{-}}^{\left(0\right)}=0$
21: **else if**$t_{h}$ is a non-removed occurrence
22: Set ${\overset{∼}{L}}_{t_{h}^{+}}^{\left(i\right)}=\omega (1-r)(k+i){\overset{∼}{L}}_{t_{h}^{-}}^{\left(i\right)}$
23: **else**$t_{h}$ is a branching event
24: Set ${\overset{∼}{L}}_{t_{h}^{+}}=\lambda {\overset{∼}{L}}_{t_{h}^{-}}$
25: **end if**
26: **end for**

We also define a slight variation of this algorithm, that we will refer to as Algorithm 1’, where no set of time points $\left(\tau_{j}\right)$ is required, and the values of ${\overset{∼}{L}}_{t}$ are not recorded through time (i.e. matrix *B* disappears). Instead, when reaching $t_{n}=t_{\mathrm{or}}$ we simply return ${\overset{∼}{L}}_{t}^{\left(0\right)}$, which by definition is an estimate of the probability density of (T,O). Note that this strategy is identical to what has been used to compute the probability density of a reconstructed phylogenetic tree under a logistic birth-death process (Leventhal et al., 2013).

These two algorithms will prove useful to deal with the general case. Furthermore, we may obtain analytical expressions for $L_{t}$ when ω=0 as well as when r=1 (Gupta et al., 2019). We reveal these in the next two subsections.

### 3.2 Special case ω=0

Suppose we can express $L_{t}^{\left(i\right)}$ as the product $L_{t}^{\left(i\right)}=u_{t}^{i}W_{t}$ where $W_{t}$ is a function of time only, and $u_{t}$ is defined as in Eq. 2.2. We first get, from the initialization in Eq. (3.2), that $W_{0}=\rho^{k_{0}}$. Moreover, substituting $u_{t}^{i}W_{t}$ in the ODE leads to

$$\begin{array}{cc} {\overset{˙}{L}}_{t}^{\left(i\right)}= & {\mathrm{iu}}_{t}^{i-1}{\overset{˙}{u}}_{t}W_{t}+u_{t}^{i}{\overset{˙}{W}}_{t}\\ = & \left(\lambda {\mathrm{iu}}_{t}^{i+1}-\gamma {\mathrm{iu}}_{t}^{i}+\mu {\mathrm{iu}}_{t}^{i-1}\right)W_{t}+u_{t}^{i}{\overset{˙}{W}}_{t}\text{.} \end{array}$$

Thus leading to the following ODE for $W_{t}$, on any epoch $\left(t_{h},t_{h+1}\right)$ where the number of sampled lineages remains fixed and equal to *k*,

$$\begin{array}{cc} u_{t}^{i}{\overset{˙}{W}}_{t}= & \left(-\gamma (i+k)u_{t}^{i}+\lambda (2k+i)u_{t}^{i+1}+\mu {\mathrm{iu}}_{t}^{i-1}-\lambda {\mathrm{iu}}_{t}^{i+1}+\gamma {\mathrm{iu}}_{t}^{i}-\mu {\mathrm{iu}}_{t}^{i-1}\right)W_{t}\\ \Rightarrow \;{\overset{˙}{W}}_{t}= & (2\lambda u_{t}-\gamma ){\mathrm{kW}}_{t}\text{.} \end{array}$$

This is very close to the ODE (2.3) governing the evolution of $p_{t}$, and it leads to (see derivation in Appendix A),

(3.9) $$\forall t\in (t_{h},t_{h+1}),\;W_{t}=W_{t_{h}}{\left(\frac{p_{t}}{p_{t_{h}}}\right)}^{k}\text{.}$$

Last, because ω=0, updates (3.3), (3.4), (3.5), (3.6), (3.7), (3.8) simplify to only the following ψ- and λ-events,

(3.10) $$\mathrm{if}\;t\;\text{is a removed leaf},\;W_{t^{+}}=\psi {\mathrm{rW}}_{t^{-}}$$

(3.11) $$\mathrm{if}\;t\;\text{is a non-removed leaf},\;W_{t^{+}}=\psi (1-r)u_{t}W_{t^{-}}$$

(3.12) $$\mathrm{if}\;t\;\text{is a sampled ancestor},\;W_{t^{+}}=\psi (1-r)W_{t^{-}}$$

(3.13) $$\mathrm{if}\;t\;\text{is a branching time},\;W_{t^{+}}=\lambda W_{t^{-}}\text{.}$$

Combining these updates with Eq. (3.9) leads to the following proposition.

Proposition 3.1 When ω=0, at any time *t* across epoch $\left(t_{h},t_{h+1}\right)$, considering that we observed so far – i.e. on $\left(0,t_{h+1}\right)$ – *v* sampled ancestors, *w* removed leaves at times $t_{j}\in W$, *x* branching events at times $t_{j}\in X$, *y* non-removed leaves at times $t_{j}\in Y$, we get,

$$\begin{array}{cc} L_{t}^{\left(i\right)}= & u_{t}^{i}W_{t}\\ \mathrm{where}\;W_{t}= & \lambda^{x}\psi^{v+w+y}{\left(1-r\right)}^{v+y}r^{w}p_{t}^{k_{t}}\underset{t_{j}\in X}{\prod }p_{t_{j}}\underset{t_{j}\in Y}{\prod }u_{t_{j}}p_{t_{j}}^{-1}\underset{t_{j}\in W}{\prod }p_{t_{j}}^{-1}\text{.} \end{array}$$

Proof We prove this proposition by induction across the epochs in Appendix E, using as the main arguments the equation updates (3.10), (3.11), (3.12), (3.13), combined with Eq. (3.9).

Note that this proposition is very similar to what is presented in Section 3 by Gupta et al. (2019). We nevertheless need to highlight two differences.

The first one is that we allow here for removal or not of the individual upon sampling, with a given probability *r*, whereas Gupta et al. (2019) considered that all individuals were removed upon sampling (r=1), and Stadler (2010) considered that individuals were not removed upon sampling (r=0).

The second difference concerns the underlying framework under which we derive our results. In Gupta et al. (2019), individuals where distinguishable (say, each one is assigned a number and they can be ordered), whereas in the present paper they are not. When individuals are ordered, the probability density $L_{t}^{\left(i\right)}$ is changed by a factor $\frac{(k+i)!}{i!}$, which is the number of ways we can arrange k+i elements in a list of size *k*, i.e. the number of ordered configurations of hidden individuals.

Note that, when reaching the origin of the tree, the formula in Proposition 3.1 reduces to a very similar formula for the probability density of T because i=0 and k=1. We summarize this as the following corollary.

Corollary 3.1.1 *When* ω=0*, the probability density of a reconstructed tree* T *with v sampled ancestors, w removed leaves at times* $t_{j}\in W$*, y non-removed leaves at times* $t_{j}\in Y$*, and branching events at times* $t_{j}\in X$*, is*

(3.14) $$P(T)=\lambda^{w+y+k_{0}-1}\psi^{v+w+y}{\left(1-r\right)}^{v+y}r^{w}\underset{t_{j}\in X\cup \{t_{\mathrm{or}}\}}{\prod }p_{t_{j}}\underset{t_{j}\in Y}{\prod }u_{t_{j}}p_{t_{j}}^{-1}\underset{t_{j}\in W}{\prod }p_{t_{j}}^{-1}$$

Proof It directly follows from Proposition 3.1, by noting that $P(T)=L_{t_{\mathrm{or}}}^{\left(0\right)}$. Note also that a rooted binary tree with $w+y+k_{0}$ leaves shows necessarily $x=w+y+k_{0}-1$ branching times.

Note that this formula is a straightforward generalization of formulas provided in Stadler (2010) (where r=0) or Stadler et al. (2011) (where ρ=0).

### 3.3 Special case r=1

When r=1, only three kinds of punctual events, corresponding to updates (3.3), (3.6), (3.8) need to be taken into account. Because the number of unsampled individuals *i* goes into formula (3.6), the simple expression $L_{t}^{\left(i\right)}=u_{t}^{i}W_{t}$ cannot be considered anymore, and one needs to find another expression. This has already been done in Gupta et al. (2019) and we only need to adapt here their result to our slightly different framework.

Proposition 3.2 When r=1, we can compute the $L_{t}^{\left(i\right)}$ values at any time *t* as

$$L_{t}^{\left(i\right)}=\overset{q}{\underset{\ell =0}{\sum }}\frac{i!}{(i-\ell )!}u_{t}^{i-\ell }W_{t}^{\left(\ell \right)}\text{.}$$

where $W_{t}$ is a *q* dimensional time-varying vector which can be computed following Algorithm 2 in Gupta et al. (2019).

Proof The proof relies on the definition of a *distinguishable version* of the probability $L_{t}^{\left(i\right)}$ as

(3.15) $${\overset{‾}{L}}_{t}^{\left(i\right)}=\frac{(k+i)!}{i!}L_{t}^{\left(i\right)}$$

which allows us to use results previously derived in Gupta et al. (2019). Details are provided in Appendix B.

Note that when there is no ω-sampling, then q=0 for all times and $W_{t}^{\left(0\right)}$ is the same as $W_{t}$ defined in the previous section.

This ends our section on the computation of $L_{t}$. It thus remains to (i) present a way to compute $M_{t}$ and (ii) combine $L_{t}$ and $M_{t}$ to get the target distribution $K_{t}$ at any time *t*. We do this in turn in the next two sections.

## 4 Calculation of $M_{t}$ – the joint density of observations above *t* and past population size

Recall that we are now interested in computing the joint density of observations above time *t* and past population size at time *t*, i.e. $\forall i\in N_{0},\;M_{t}^{\left(i\right)}≔P(T_{t}^{↑},O_{t}^{↑},I_{t}=k_{t}+i)$. We start by presenting the ODEs satisfied by $M_{t}$, before turning to its resolution for specific parameter sets. The approach is very similar to the one presented in the previous section to compute $L_{t}$, with the slight difference that we will need to traverse the tree forward in time instead of backward in time.

### 4.1 Set of ODEs satisfied by $M_{t}$

At the time of origin of the process $t_{\mathrm{or}}$, we only observe one starting lineage in $T_{t_{\mathrm{or}}}^{↑}$. This provides us with the following initialization condition on *M*,

$$M_{t_{\mathrm{or}}}^{\left(i\right)}=P(I_{t_{\mathrm{or}}}=1+i)=1_{i=0}\text{.}$$

We then derive the ODEs driving the evolution of $M_{t}$ across an epoch on which the number of observed lineages is fixed and equal to *k*. Suppose we know $M_{t}$, and we observe no punctual event on the infinitesimal time interval (t-δt,t). Unobservable events have already been illustrated in Fig. 2. It allows us to get

$$M_{t-\delta t}^{\left(i\right)}=\left(1-\gamma (i+k)\delta t\right)M_{t}^{\left(i\right)}+\lambda (2k+i-1)\delta t1_{i>0}M_{t}^{\left(i-1\right)}+\mu (i+1)\delta {\mathrm{tM}}_{t}^{\left(i+1\right)}\text{.}$$

Subtracting $M_{t}^{\left(i\right)}$ from both sides, multiplying by -1,dividing by δt and letting δt→0, we get the following set of ODEs driving the evolution of $M_{t}$,

(4.1) $$\begin{array}{cc} \forall i\in N_{0},\;M_{t_{\mathrm{or}}}^{\left(i\right)}= & 1_{i=0}\\ {\overset{˙}{M}}_{t}^{\left(i\right)}= & \gamma (i+k)M_{t}^{\left(i\right)}-\lambda (2k+i-1)1_{i>0}M_{t}^{\left(i-1\right)}-\mu (i+1)M_{t}^{\left(i+1\right)}\text{.} \end{array}$$

Last, we need to take into account the evolution of $M_{t}$ at punctual events. Again, there are 6 types of punctual events that we can come across at time *t* in the past, listed below and illustrated in Fig. 3. We denote $M_{t^{-}}$ the probability just after (i.e. below) the punctual event and $M_{t^{+}}$ the probability immediately before (i.e. up). Because we are here deriving $M_{t}$ forward in time, one needs to carefully note differences with results derived in Section 3 relating to the number of lineages before and after the event. We can indeed find the same punctual events, namely,

1. a leaf of $T_{t}^{↓}$, labeled as removed. This is a ψ-sampling with removal event for which the number of sampled lineages decreases by one and the number of unsampled lineages remains unchanged. This gives,
  (4.2) $$M_{t^{-}}^{\left(i\right)}=\psi {\mathrm{rM}}_{t^{+}}^{\left(i\right)}\text{.}$$
2. a leaf of $T_{t}^{↓}$, labeled as non-removed. This is a ψ-sampling without removal event for which one sampled lineages becomes unsampled. This gives,
  (4.3) $$M_{t^{-}}^{\left(i\right)}=\psi (1-r)1_{i>0}M_{t^{+}}^{\left(i-1\right)}\text{.}$$
3. a sampled ancestor along a branch of $T_{t}^{↓}$, necessarily labeled as non-removed. This is a ψ-sampling without removal event which does not affect the number of lineages. It gives,
  (4.4) $$M_{t^{-}}^{\left(i\right)}=\psi (1-r)M_{t^{+}}^{\left(i\right)}\text{.}$$
4. an occurrence in $O_{t}^{↓}$, labeled as removed. This is a ω-sampling with removal event, for which the number of unsampled lineages decreases by one. This gives,
  (4.5) $$M_{t^{-}}^{\left(i\right)}=\omega r(i+1)M_{t^{+}}^{\left(i+1\right)}\text{.}$$
5. an occurrence in $O_{t}^{↓}$, labeled as non-removed. This is a ω-sampling without removal event which does not affect the number of lineages. It gives,
  (4.6) $$M_{t^{-}}^{\left(i\right)}=(k+i)\omega (1-r)M_{t^{+}}^{\left(i\right)}\text{.}$$
6. a branching event between two branches of $T_{t}^{↓}$.
  This is a λ-event increasing the number of sampled lineages by one. This gives,
  (4.7) $$M_{t^{-}}^{\left(i\right)}=\lambda M_{t^{+}}^{\left(i\right)}\text{.}$$

Finally, upon reaching present time 0, one needs to take into account the ρ-sampling, leading to the following update,

(4.8) $$M_{0^{-}}^{\left(i\right)}={\left(1-\rho \right)}^{i}\rho^{k_{0}}M_{0^{+}}^{\left(i\right)}\text{.}$$

Note, as for $L_{t}$, that these updates can be adapted to the case when we do not observe the removal status of individuals. The update corresponding to a leaf of T is the sum of updates (4.2), (4.3), the update corresponding to an occurrence event is the the sum of updates (4.5), (4.6), while updates (4.4), (4.7) are unchanged.

As already exhibited for $L_{t}$, we can build a similar algorithm to compute $M_{t}$ in the general case, relying on a numerical ODE solver for approximating Eq. (4.1). As for Algorithm 1’ previously introduced to compute the probability density of (T,O), a slight variation of this algorithm would allow one to compute an estimate of the probability density of (T,O) by summing the $M_{0}^{\left(i\right)}$’s over all *i*. Note that this strategy is identical to what has been used to compute the probability density of a reconstructed phylogenetic tree under a logistic birth-death process (Etienne et al., 2012, Laudanno et al., 2020).

While this approach is in theory a good approximation, it requires fixing arbitrarilly a truncation parameter *N*, and exponentiating matrices of dimension N×N, leading to potential speed or accurracy issues. In the remainder of this section, we derive analytical results to avoid resorting to a numerical ODE solver in specific cases.

### 4.2 The corresponding generating function

We introduce now the generating function corresponding to the density $M_{t}$, which will prove useful to get analytical results,

M^(t,z)≔∑i=0∞ziMt(i).

The initial condition on *M* translates into, ∀z,M^(tor,z)=1. The ODE (4.1) furthermore translates into the following partial differential equation (PDE),

∂tM^=∑i=0∞ziγ(i+k)Mt(i)-λ(2k+i-1)1i>0Mt(i-1)-μ(i+1)Mt(i+1)=γk∑i=0∞ziMt(i)+γ∑i=1∞iziMt(i)-λ∑i=0∞zi+1(2k+i)Mt(i)-μ∑i=1∞izi-1Mt(i)=γkM^+γz∂zM^-2kλzM^-λz2∂zM^-μ∂zM^=-k(2λz-γ)M^-(λz2-γz+μ)∂zM^.

Our target generating function M^ is thus the solution of the following PDE problem across a given epoch $\left(t_{h-1},t_{h}\right)$, on which the number of observed lineages remains constant and equal to *k*,

(4.9) M^(th,z)=F(z)∂tM^+(λz2-γz+μ)∂zM^+k(2λz-γ)M^=0.

Solving this PDE problem allows us to obtain an analytical expression of M^ for any time across an epoch, provided we know the expression of M^(th,z) at the end of the epoch.

Proposition 4.1 The solution to the PDE problem (4.9) is given by

M^(t,z)=Fu(th-t,z)R(th-t,z)k

where we introduce R(t,z)=p(t,z)/(1-z) to ease the notation.

Proof We used the method of characteristics to solve this first order linear PDE, see derivations in Appendix C.

Between epochs, one must also update M^ according to punctual events taking place. Previously presented updates of *M* (Eqs. (4.2), (4.3), (4.4), (4.5), (4.6), (4.7)) translate into the following updates for M^,

if t is a removed leaf,

(4.10) M^(t-,z)=∑i=0∞ziψrMt+(i)=ψrM^(t+,z)

if *t* is a non-removed leaf,

(4.11) M^(t-,z)=∑i=0∞ziψ(1-r)1i>0Mt+(i-1)=ψ(1-r)zM^(t+,z)

if *t* is a sampled ancestor,

(4.12) M^(t-,z)=∑i=0∞ziψ(1-r)Mt+(i)=ψ(1-r)M^(t+,z)

if *t* is a removed occurrence,

(4.13) M^(t-,z)=∑i=0∞ziωr(i+1)Mt+(i+1)=ωr∂zM^(t+,z)

if *t* is a non-removed occurrence,

(4.14) M^(t-,z)=∑i=0∞ziω(1-r)(k+i)Mt+(i)=ω(1-r)kM^(t+,z)+z∂zM^(t+,z)

if *t* is a branching event,

(4.15) M^(t-,z)=∑i=0∞ziλMt+(i)=λM^(t+,z).

If we are interested in the distribution at some point, we can thus start the formula at $t_{\mathrm{or}}$ with F(z)=1, and then iteratively alternate between the updates at punctual events and the use of Proposition 4.1 over each epoch. When reaching present time 0, the step of ρ-sampling expressed in Eq. (4.8) moreover translates into,

(4.16) M^(0-,z)=∑i=0∞zi(1-ρ)iρk0M0+(i)=ρk0M^(0+,(1-ρ)z).

While this procedure in theory allows us to get the analytical formula of M^ at any time, updates (4.13), (4.14) require differentiating the generating function, greatly complicating the expression of the function after a few occurrences. When ω=0, these two updates disappear and a nice recursion leads to a closed-form formula that we will detail in Proposition 4.3.

We implemented this procedure in the *SageMath* programming language able to deal with symbolic calculus. We were however not able to make it find concise expressions, and computing these successive derivatives was too time-consuming to be applicable to standard datasets in the field. Instead, when ω≠0.

We suggest another strategy for computing the $M_{t}^{\left(i\right)}$’s, namely approximating M^ across punctual events by a polynomial of order *N* , $\sum_{l=0}^{N}{\overset{∼}{M}}_{t}^{\left(l\right)}z^{l}$, while still relying on Proposition 4.1 to drive the evolution of the probability generating function between events. This is a more efficient alternative to numerically solving the ODE system. We only need to derive the expression of the generating function at punctual events as given in the following Proposition 4.2.

Proposition 4.2 The derivatives in z=0 of a generative function which can be expressed as

M^(th-t,z)≔R(th-t,z)k∑l=0NM∼th(l)u(th-t,z)l

can be numerically computed using the formula

∂ziM^(th-t,z)z=0=Δλ2e-Δ(th-t)k∑α=0i∑l=αNM∼th(l)iαl!(l-α)!∏m=0i-α-1(2k+l+m)x1x2l-α-x1+x2e-Δ(th-t)α1-e-Δ(th-t)l+i-2αx2-x1e-Δ(th-t)-(2k+l+i-α).

Proof The derivation is detailed in Appendix D.1.

This derivation is at the heart of Algorithm 2, allowing to follow the evolution of the ${\overset{∼}{M}}_{t}^{\left(i\right)}$’s through each epoch, as well as at times when we want to record them.

We will refer to Algorithm 2’ as the slight variation of this algorithm aimed at computing the density of (T,O). No set of time points $\left(\tau_{j}\right)$ is required, and the values of ${\overset{∼}{M}}_{t}$ are not recorded through time (i.e. matrix B′ disappears). Instead, when reaching $t_{h}=t_{0}$ we simply return $\sum_{i=0}^{N}\rho^{k_{0}}{\left(1-\rho \right)}^{i}{\overset{∼}{M}}^{\left(i\right)}$.

**Table:** 

**Algorithm 2:** Computes a numerical approximation of Mt for a specic set of times
**Input**: Observed tree and occurrence data (T,O),
parameters $\left(t_{\mathrm{or}},\lambda ,\mu ,\psi ,\omega ,\rho \right)$,
set of time points ${\left(\tau_{j}\right)}_{j=1}^{S}$ for which we want to compute the density,
and the truncation *N* setting the accuracy of the algorithm.
**Output**: A numerical approximation of $M_{t}$ at times ${\left(\tau_{j}\right)}_{j=1}^{S},{\left({\overset{∼}{M}}_{\tau_{j}}^{\left(i\right)}\right)}_{\begin{array}{c} i\in \{0,1,\ldots ,N\}\\ j\in \{1,2,\ldots ,S\} \end{array}}$.
1: Pool all $\left(\tau_{j}\right)$ and all branching and sampling times of (T,O) in an ordered list ${\left(t_{h}\right)}_{h=1}^{n}$
2: Set j=S and $B^{\prime }$ as a S×(N+1) empty matrix
3: Set $\forall i\in \{0,1,\ldots ,N\},{\overset{∼}{M}}^{\left(i\right)}=1_{i=0}$
4: Set k=1
5: **for**h=n-1,n-2,…,0**do**
6: Compute the values right before the punctual event,
$$\begin{array}{cc} {\overset{∼}{\overset{∼}{M}}}^{\left(i\right)}= & {\left(\frac{\Delta }{\lambda^{2}}e^{-\sqrt{\Delta }(t_{h}-t)}\right)}^{k}\overset{i}{\underset{\alpha =0}{\sum }}\overset{N}{\underset{l=\alpha }{\sum }}{\overset{∼}{M}}_{t_{h}}^{\left(l\right)}\left(\begin{array}{c} l\\ \alpha \end{array}\right)\frac{1}{(i-\alpha )!}\left(\overset{i-\alpha -1}{\underset{m=0}{\prod }}(2k+l+m)\right)\\ & {\left(-x_{1}+x_{2}e^{-\sqrt{\Delta }(t_{h}-t)}\right)}^{\alpha }{\left(x_{1}x_{2}\right)}^{l-\alpha }{\left(1-e^{-\sqrt{\Delta }(t_{h}-t)}\right)}^{l+i-2\alpha }{\left(x_{2}-x_{1}e^{-\sqrt{\Delta }(t_{h}-t)}\right)}^{-(2k+l+i-\alpha )} \end{array}$$
7: **if**$t_{h}=\tau_{j}$**then**
8: Record the result in $B^{\prime }$ : $\forall i,{B^{\prime }}^{\left(j,i\right)}={\overset{∼}{\overset{∼}{M}}}^{\left(i\right)}$
9: Set j=j-1.
10: **end if**
11: **if**$t_{h}=0$ or $t_{h}=\tau_{S}$
12: **return**$B^{\prime }$
13: **els if**$t_{h}$ is a removed leaf
14: Update $\forall i,\;{\overset{∼}{M}}^{\left(i\right)}=\psi r{\overset{\sim }{\overset{∼}{M}}}^{\left(i\right)}$
15: Set k=k-1
16: **else if**$t_{h}$ is a non-removed leaf
17: Update ${\overset{∼}{M}}^{\left(0\right)}=0$ and $\forall i>0,\;{\overset{∼}{M}}^{\left(i\right)}=\psi (1-r){\overset{\sim }{\overset{∼}{M}}}^{\left(i-1\right)}$
18: Set k=k-1
19: **else if**$t_{h}$ is a sampled ancestor
20: Update $\forall i,\;{\overset{∼}{M}}^{\left(i\right)}=\psi (1-r){\overset{\sim }{\overset{∼}{M}}}^{\left(i\right)}$
21: **else if**$t_{h}$ is a removed occurrence
22: Update $\forall i<N,\;{\overset{∼}{M}}^{\left(i\right)}=\omega r(i+1){\overset{\sim }{\overset{∼}{M}}}^{\left(i+1\right)}$ and ${\overset{∼}{M}}^{\left(N\right)}=0$
23: **else if**$t_{h}$ is a non-removed occurrence
24: Update $\forall i,\;{\overset{∼}{M}}^{\left(i\right)}=\omega (1-r)(k+i){\overset{\sim }{\overset{∼}{M}}}^{\left(i\right)}$
25: **else**$t_{h}$ is a branching event
26: Update $\forall i,\;{\overset{∼}{M}}^{\left(i\right)}=\lambda {\overset{\sim }{\overset{∼}{M}}}^{\left(i\right)}$
27: Set k=k+1
28: **end if**
29: **end for**

Note that we tried to follow an analogous generating function approach as an alternative to Algorithm 1 to compute $L_{t}$ as well. This leads to another PDE problem, described in Appendix F, that will require further work to be solved.

### 4.3 Special case ω=0

We were not able to come with any analytical simplification, as in the previous section, for the case r=1. However, for the special case ω=0, corresponding to the special case leading to the observation of O=∅, a nice recursion leads to a closed-form formula for M^.

Proposition 4.3 When ω=0, at any time *t*, considering that we have observed so far –i.e. on $\left(t,t_{\mathrm{or}}\right)$ – *v* sampled ancestors, *w* removed leaves at times $t_{j}\in W$, *x* branching events at times $t_{j}\in X$, *y* non-removed leaves at times $t_{j}\in Y$, we get,

M^(t,z)=λxψv+w+yrw(1-r)v+y∏tj∈X∪{tor}R(tj-t,z)∏tj∈WR(tj-t,z)-1∏tj∈Yu(tj-t,z)R(tj-t,z)-1.

Proof We prove this result by induction across the epochs of T in Appendix E, using as the main arguments the update Eqs. (4.10), (4.11), (4.12), (4.15), combined with Proposition 4.1 driving the evolution across an epoch.

As a simple corollary of this result, when $t_{h}=0$ is the present, we get back the same probability density formula of T as provided, e.g. in Theorem 3.5 in Stadler (2010) (when r=0), in Section 3 in Gupta et al. (2019) (when r=1), or in our previous Corollary 3.1.1.

Indeed, Proposition 4.3 offers yet another proof of Corollary 3.1.1 by noting that

P(T)=∑i=0∞M0-(i)=M^(0-,1)=ρk0M^(0+,1-ρ)

where the last equality follows from Eq. (4.16) taking into account the ρ-sampling at present. Note that this alternative proof is also presented in (Laudanno et al., 2020).

When ω=0, Proposition 4.3 also offers an alternative to Algorithm 2 for deriving $M_{t}$. Indeed, resorting to the generating function to get back the probability density, one can get the following corollary.

Corollary 4.3.1 *When* ω=0*, at any time t, considering that we have observed so far –i.e. on* $\left(t,t_{\mathrm{or}}\right)$ *– v sampled ancestors, w removed leaves at times* $t_{j}\in W$*, x branching events at times* $t_{j}\in X$*, y non-removed leaves at times* $t_{j}\in Y$*, we can compute* $M_{t}^{\left(i\right)}$ *using the following recursion,*

$$\begin{array}{cc} M_{t}^{\left(0\right)}= & \lambda^{x}\psi^{v+w+y}r^{w}{\left(1-r\right)}^{v+y}\underset{t_{j}\in X\cup \{t_{\mathrm{or}}\}}{\prod }R(t_{j}-t,0)\underset{t_{j}\in W}{\prod }R{\left(t_{j}-t,0\right)}^{-1}\underset{t_{j}\in Y}{\prod }u(t_{j}-t,0)R{\left(t_{j}-t,0\right)}^{-1}\\ M_{t}^{\left(i\right)}= & \frac{1}{i}\overset{i}{\underset{\alpha =1}{\sum }}M_{t}^{\left(i-\alpha \right)}C^{\left(\alpha \right)} \end{array}$$

*where we define*

$$\begin{array}{cc} C^{\left(\alpha \right)}= & 2\underset{t_{j}\in X\cup \{t_{\mathrm{or}}\}}{\Pi }a_{t_{j}-t}^{\alpha }-2\underset{t_{j}\in W}{\Pi }a_{t_{j}-t}^{\alpha }-\underset{t_{j}\in Y}{\Pi }(a_{t_{j}-t}^{\alpha }+b_{t_{j}-t}^{\alpha })\\ a_{t}= & \left(1-e^{-\sqrt{\Delta }t}\right){\left(x_{2}-x_{1}e^{-\sqrt{\Delta }t}\right)}^{-1}\\ b_{t}= & \left(x_{1}-x_{2}e^{-\sqrt{\Delta }t}\right){\left(x_{1}x_{2}-x_{2}x_{1}e^{-\sqrt{\Delta }t}\right)}^{-1}\text{.} \end{array}$$

Proof The probability density $M_{t}^{\left(i\right)}$ can be found back by taking

Mt(i)=1i!∂ziM^(t,z)z=0.

The result follows from the derivation of these derivatives in Appendix D.2.

This special case ends the section. In the next section, we will combine results from Sections 3 and 4 and use our ability to compute $L_{t}$ and $M_{t}$ to compute $K_{t}$, the probability distribution of the population size given (T,O).

## 5 The distribution of past population size conditioned on observations

### 5.1 The distribution at fixed times

In Section 3, we explained how to compute $L_{t}$, the probability density of the observations below time *t* conditioned on the population size at time *t*. This relies either on Algorithm 1 in the general case, or on the more optimized Proposition 3.1 in case ω=0, or Proposition 3.2 in the case r=1.

In Section 4, we explained how to compute $M_{t}$, the probability density of the observations above time *t* and the population size at time *t*. This relies either on Algorithm 2 in the general case, or on the more optimized Corollary 4.3.1 when ω=0. We now combine $L_{t}$ and $M_{t}$ to derive the probability distribution of the population size given (T,O). Provided we have stored numerical values ${\left({\overset{∼}{L}}_{\tau_{j}}^{\left(i\right)}\right)}_{\begin{array}{c} i\in \{0,1,\ldots ,N\}\\ j\in \{1,2,\ldots ,S\} \end{array}}$ and ${\left({\overset{∼}{M}}_{\tau_{j}}^{\left(i\right)}\right)}_{\begin{array}{c} i\in \{0,1,\ldots ,N\}\\ j\in \{1,2,\ldots ,S\} \end{array}}$ for a set of time points ${\left(\tau_{j}\right)}_{j=1}^{S}$, recall from the first section that we obtain

$$\begin{array}{cc} K_{\tau_{j}}^{\left(i\right)}= & P\left(I_{\tau_{j}}=k_{\tau_{j}}+i|T,O\right)\\ = & \frac{L_{\tau_{j}}^{\left(i\right)}M_{\tau_{j}}^{\left(i\right)}}{P(T,O)}\\ \approx & \frac{{\overset{∼}{L}}_{\tau_{j}}^{\left(i\right)}{\overset{∼}{M}}_{\tau_{j}}^{\left(i\right)}}{P(T,O)}\mathrm{if}i⩽N,\;\mathrm{and}\;0\;\mathrm{otherwise}\text{.} \end{array}$$

Note that the denominator needs only be computed once, by evaluating $\sum_{i=0}^{N}{\overset{∼}{L}}_{\tau_{j}}^{\left(i\right)}{\overset{∼}{M}}_{\tau_{j}}^{\left(i\right)}$ for example at time $\tau_{j}=t_{\mathrm{or}}$ or $\tau_{j}=0$ as described in previous sections.

**Fig. 4:**
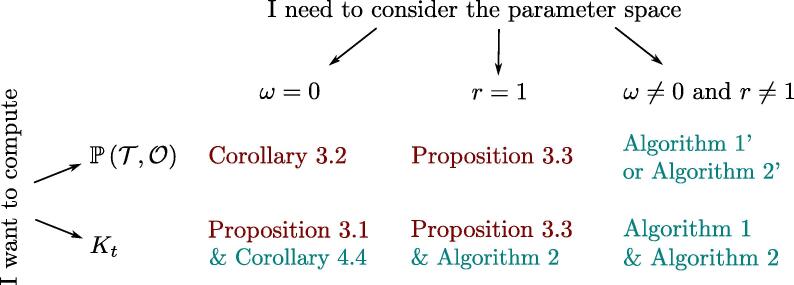
The most efficient results depending on the parameter space considered. In red, results already described in Stadler (2010) and Gupta et al. (2019). In blue, the new contribution of this manuscript. (For interpretation of the references to colour in this figure legend, the reader is referred to the web version of this article.)

Depending on the parameter space that one wants to consider, it thus remains to arrange pieces stemming from the previous sections. We provide a flowchart in Fig. 4 to guide the reader to chose the most efficient path.

### 5.2 Generator of trajectories

The previous result gives us the distribution of the population size at any time in the past, but does not state anything about population size trajectories. We provide now an approximate way of simulating population size trajectories conditioned on (T,O).

Indeed, recall we have,

$$\begin{array}{cc} K_{t}^{\left(i\right)}≔ & P(I_{t}=k_{t}+i|T,O)\propto L_{t}^{\left(i\right)}M_{t}^{\left(i\right)}\\ {\overset{˙}{L}}_{t}^{\left(i\right)}= & -\gamma (k_{t}+i)L_{t}^{\left(i\right)}+\lambda (2k_{t}+i)L_{t}^{\left(i+1\right)}+\mu {\mathrm{iL}}_{t}^{\left(i-1\right)}\\ {\overset{˙}{M}}_{t}^{\left(i\right)}= & \gamma (k_{t}+i)M_{t}^{\left(i\right)}-\mu (i+1)M_{t}^{\left(i+1\right)}-\lambda (2k_{t}+i-1)1_{i>0}M_{t}^{\left(i-1\right)}\text{.} \end{array}$$

We thus get,

(5.1) $$\begin{array}{cc} {\overset{˙}{K}}_{t}^{\left(i\right)}\propto & {\overset{˙}{L}}_{t}^{\left(i\right)}M_{t}^{\left(i\right)}+L_{t}^{\left(i\right)}{\overset{˙}{M}}_{t}^{\left(i\right)}\\ \propto & -\gamma (k_{t}+i)L_{t}^{\left(i\right)}M_{t}^{\left(i\right)}+\lambda (2k_{t}+i)L_{t}^{\left(i+1\right)}M_{t}^{\left(i\right)}+\mu {\mathrm{iL}}_{t}^{\left(i-1\right)}M_{t}^{\left(i\right)}\\ + & \gamma (k_{t}+i)M_{t}^{\left(i\right)}L_{t}^{\left(i\right)}-\mu (i+1)M_{t}^{\left(i+1\right)}L_{t}^{\left(i\right)}-\lambda (2k_{t}+i-1)1_{i>0}M_{t}^{\left(i-1\right)}L_{t}^{\left(i\right)}\\ \propto & \lambda (2k_{t}+i)\frac{L_{t}^{\left(i+1\right)}}{L_{t}^{\left(i\right)}}K_{t}^{\left(i\right)}+\mu i\frac{L_{t}^{\left(i-1\right)}}{L_{t}^{\left(i\right)}}K_{t}^{\left(i\right)}-\lambda (2k_{t}+i-1)1_{i>0}\frac{L_{t}^{\left(i\right)}}{L_{t}^{\left(i-1\right)}}K_{t}^{\left(i-1\right)}-\mu (i+1)\frac{L_{t}^{\left(i\right)}}{L_{t}^{\left(i+1\right)}}K_{t}^{\left(i+1\right)}\\ \propto & Q_{t}^{\left(i,i\right)}K_{t}^{\left(i\right)}+Q_{t}^{\left(i-1,i\right)}K_{t}^{\left(i-1\right)}+Q_{t}^{\left(i+1,i\right)}K_{t}^{\left(i+1\right)}\text{.} \end{array}$$

We introduced in the last line the following notation,

$$\begin{array}{cc} Q_{t}^{\left(i+1,i\right)}= & -\mu (i+1)\frac{L_{t}^{\left(i\right)}}{L_{t}^{\left(i+1\right)}}\\ Q_{t}^{\left(i-1,i\right)}= & -\lambda (2k_{t}+i-1)1_{i>0}\frac{L_{t}^{\left(i\right)}}{L_{t}^{\left(i-1\right)}}\\ Q_{t}^{\left(i,i\right)}= & \lambda (2k_{t}+i)\frac{L_{t}^{\left(i+1\right)}}{L_{t}^{\left(i\right)}}+\mu i\frac{L_{t}^{\left(i-1\right)}}{L_{t}^{\left(i\right)}}\text{.} \end{array}$$

Using these, we see that $Q_{t}^{\left(i,i\right)}=-\left(Q_{t}^{\left(i,i+1\right)}+Q_{t}^{\left(i,i-1\right)}\right)$. This allows us to draw trajectories of the number of ancestors in the past as a time-continuous Markov process with the (inhomogeneous) rates $Q_{t}$ written above.

Observe that we could equally write these ODE coefficients using the $M_{t}^{\left(i\right)}$’s. This gives,

(5.2) $$\begin{array}{cc} {\overset{˙}{K}}_{t}^{\left(i\right)}\propto & \lambda (2k_{t}+i)\frac{M_{t}^{\left(i\right)}}{M_{t}^{\left(i+1\right)}}K_{t}^{\left(i+1\right)}+\mu i\frac{M_{t}^{\left(i\right)}}{M_{t}^{\left(i-1\right)}}K_{t}^{\left(i-1\right)}-\mu (i+1)\frac{M_{t}^{\left(i+1\right)}}{M_{t}^{\left(i\right)}}K_{t}^{\left(i\right)}-\lambda (2k_{t}+i-1)1_{i>0}\frac{M_{t}^{\left(i-1\right)}}{M_{t}^{\left(i\right)}}K_{t}^{\left(i\right)}\\ \propto & R_{t}^{\left(i+1,i\right)}K_{t}^{\left(i+1\right)}+R_{t}^{\left(i-1,i\right)}K_{t}^{\left(i-1\right)}+R_{t}^{\left(i,i\right)}K_{t}^{\left(i\right)} \end{array}$$

where we introduced in the last line the following notation,

$$\begin{array}{cc} R_{t}^{\left(i+1,i\right)}= & \lambda (2k_{t}+i)\frac{M_{t}^{\left(i\right)}}{M_{t}^{\left(i+1\right)}}\\ R_{t}^{\left(i-1,i\right)}= & \mu i\frac{M_{t}^{\left(i\right)}}{M_{t}^{\left(i-1\right)}}\\ R_{t}^{\left(i,i\right)}= & -\lambda (2k_{t}+i-1)1_{i>0}\frac{M_{t}^{\left(i-1\right)}}{M_{t}^{\left(i\right)}}-\mu (i+1)\frac{M_{t}^{\left(i+1\right)}}{M_{t}^{\left(i\right)}}\text{.} \end{array}$$

This is a standard result for Markov chains that are conditioned on a final state, and the shape of the newly derived transition kernel is called a Doob’s transform (Levin and Peres, 2017). Note that these transitions symplify for special cases when we have an analytical expression of either $L_{t}^{\left(i\right)}$ or $M_{t}^{\left(i\right)}$.

### 5.3 Numerical implementation

Results of this paper have been implemented numerically and the code is freely available on GitLab: *https://gitlab.com/MMarc/popsize-distribution/*.

We used the numerical implementation to verify the correctness of the results in several ways:

1. We verified that the values of the probability density of (T,O) computed using $L_{t}$ and $M_{t}$ (i.e. respectively using Algorithms 1’ and 2’) were equivalent to values computed using already known formulas when (ω=0,r=0) (Stadler, 2010) or when r=1 (Gupta et al., 2019). See result in Fig. 5AB.

**Fig. 5:**
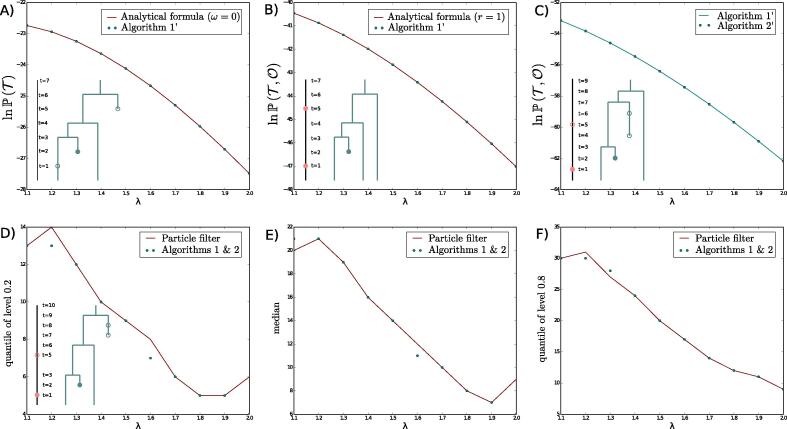
Assessment of the accuracy of the methods presented in this paper, on toy datasets. First row, probability density of data, A) against known analytical formula when ω=0 and (μ,ρ,ψ,r)=(1,0.5,0.3,0.2); B) against known analytical formula when r=1 and (μ,ρ,ψ,ω)=(1,0.5,0.3,0.6); C) obtained using Algorithms 1’ or 2’ otherwise, with (μ,ρ,ψ,r,ω)=(1,0.5,0.3,0.2,0.6). Second row, quantiles of the population size distribution, against the particle filter in Vaughan et al. (2019), with parameters (μ,ρ,ψ,r,ω)=(1,0.1,0.001,0.5,0.001). D) quantile of level 0.2; E) median; F) quantile of level 0.8.
2. We verified that the values of the probability density of (T,O) computed using $L_{t}$ or $M_{t}$ (Algorithms 1’ and 2’) were identical on examples for which no previous formula was known. See result in Fig. 5C.
3. We assessed the distribution of the population size against the only numerical method performing the same goal, the particle filtering developed in Vaughan et al. (2019). We compared values of a few quantiles computed using the two methods, see result in Fig. 5DEF). Note that (Vaughan et al., 2019) considered that we never have data on the removal status of individuals. We thus adapted our developments to this scenario in this specific comparison, by summing updates corresponding to the removal or not of the sampled individuals.

On each of these sanity checks, we verified that different quantities match across different λ values. Note that we could equivalently have chosen any other parameter to be varied.

**Fig. 6:**
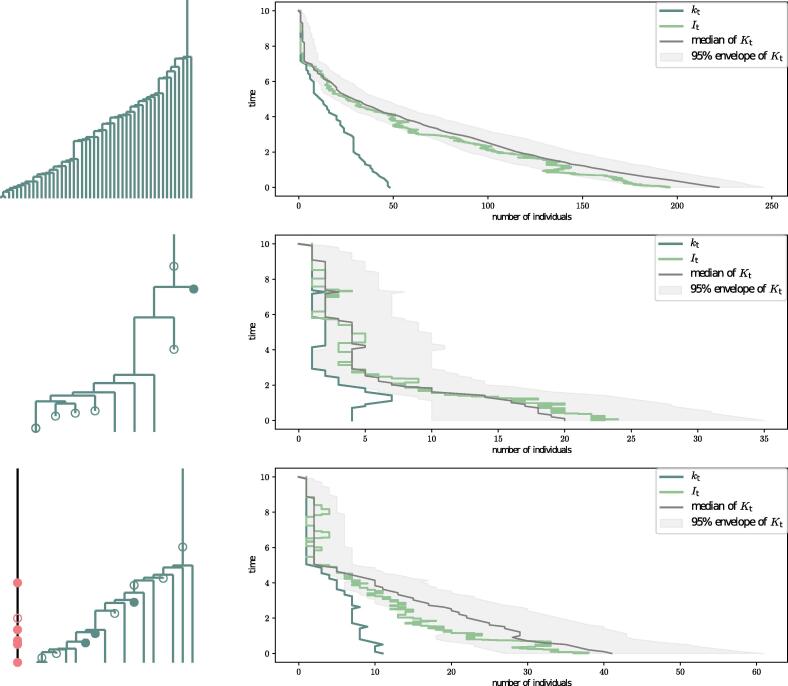
Inferred population size distribution $K_{t}$ using (T,O) matches the simulated population size trajectory $I_{t}$ under three different processes: A) A homogeneous birth-death with ρ-sampling at present; B) A homogeneous birth-death with ρ-sampling at present and ψ-sampling through time; C) A homogeneous birth-death process with ρ-, ψ- and ω-sampling. Note that we plot on the same graph $k_{t}$, the number of observed lineages in the tree, as this is an obvious lower bound in our population size inference.

We also illustrate in Fig. 6 our target distribution $K_{t}$ of the past population size conditioned on (T,O), on a few simulated examples.

## 6 Discussion

The results we have derived in this paper fit into two main categories. The first category concerns results allowing one to compute the probability density of a tree and occurrences, while the second category concerns results allowing one to compute the probability distribution of the population size in the past. We discuss these two categories below, before presenting ideas for future extensions of the model.

### 6.1 Using the probability density of the data

We present in this article new ways to compute the probability density of the data, P(T,O). For the special cases (ω=0,r=0) or (r=1), efficient calculations are available in Stadler, 2010, Gupta et al., 2019. Our two Algorithms 1’ and 2’ have the potential to improve the computation time of P(T,O) also when ω≠0 and r≠1. When analysing data, as described below, often this probability density is conditioned on sampling at least one individual, using $u_{t_{\mathrm{or}}}$ (Stadler, 2012).

In the case that the tree is known, we can use $P(T,O|\lambda ,\mu ,\rho ,\psi ,r,\omega ,t_{\mathrm{or}})$ (with conditioning on sampling at least one individual) to obtain maximum likelihood parameter estimates for the birth-death parameters λ,μ as well as the sampling parameters ρ,ψ,r,ω. For special cases of this model, it has been shown that not all sampling parameters are identifiable (see e.g. Stadler et al., 2019). Future work will involve investigating which of the sampling parameters in the general model can be estimated.

On the other hand, data may consist of sequencing data A and occurrence data O. Bayesian tools are then typically employed to obtain a sample from the posterior distribution of the parameters using Markov chain Monte Carlo methods. The posterior distribution is,

$$f(T,\theta ,\lambda ,\mu ,\rho ,\psi ,\omega ,t_{\mathrm{or}}|O,A)\propto f(A|T,\theta )f(O,T|\lambda ,\mu ,\rho ,\psi ,r,\omega ,t_{\mathrm{or}})f(\lambda ,\mu ,\rho ,\psi ,r,\omega ,\theta ,t_{\mathrm{or}}),$$

with θ summarizing the parameters of the model of molecular evolution and $f(\lambda ,\mu ,\rho ,\psi ,r,\omega ,\theta ,t_{\mathrm{or}})$ being the prior distribution on the model parameters.

### 6.2 Probability distribution of past population sizes

The main results of this paper allow oneto compute the probability distribution of the population size in the past and to generate population size trajectories conditioned on (T,O) (Section 5).

Given a tree and occurrences together with birth-death parameters (which may be the maximum likelihood parameters obtained based on the tree and record of occurrences), we can simulate the distribution of past population sizes as described in Section 5.2. Furthermore, we can calculate the probability of a population size at any time in the past as described in Section 5.1.

If we are instead provided with sequencing data A and occurrence data O, and want to generate a simulated ensemble characterizing the posterior distribution of past population size trajectories I, we can use the following strategy. The posterior distribution is,

$$f(T,I,\theta ,\lambda ,\mu ,\rho ,\psi ,\omega ,t_{\mathrm{or}}|O,A)=f(I|T,\theta ,\lambda ,\mu ,\rho ,\psi ,\omega ,t_{\mathrm{or}},O,A)f(T,\theta ,\lambda ,\mu ,\rho ,\psi ,\omega ,t_{\mathrm{or}}|O,A)$$

We have described above how to obtain a sample from the posterior distribution $f(T,\;\theta ,\;\lambda ,\;\mu ,\;\rho ,\;\psi ,\;\omega ,\;t_{\mathrm{or}}|O,\;A)$ using Markov chain Monte Carlo. For each sample of $(T,\;\theta ,\;\lambda ,\;\mu ,\;\rho ,\;\psi ,\;\omega ,\;t_{\mathrm{or}})$ thus obtained, we can simulate an appropriately conditioned population size trajectory I as described in Section 5.2. The ensemble of trajectories thus generated has the required distribution. We can employ an analogous procedure if we are interested in the posterior probability distribution of the population size at a particular time *t*. For each posterior sample of $(T,\theta ,\lambda ,\mu ,\rho ,\psi ,\omega ,t_{\mathrm{or}})$, we can calculate the population size distribution at time *t* using Section 5.1. The posterior population size at time *t* is then the average over all these conditional distributions.

### 6.3 Increased efficiency opens new research avenues

Both the density P(T,O) and the probability distribution of the population size in the past $\left(K_{t}\right)$ can be obtained using the Monte-Carlo particle filtering algorithm developed in Vaughan et al. (2019). The new approach presented in this paper is nevertheless appealing for two reasons. First, it provides a direct link with previous analytical formulas developed in Stadler, 2010, Gupta et al., 2019, thus improving our understanding of these processes and leading to very efficient results in the specific case where ω=0. Second, Algorithms 1 and 2 have the potential to be more efficient alternatives to the Monte-Carlo particle filtering algorithm. Computing quantiles shown in Fig. 5DEF using the particle filtering took a few days, as compared to a few minutes with our method, mainly because it can be applied directly on a fixed tree and does not need to be part of a MCMC. A more thorough quantitative comparison of both approaches would require to implement this work in a MCMC framework, which is beyond the scope of this paper.

This increased efficiency could open up the possibility to analyse much bigger datasets in the near future. In macroevolution, the study of clades with a huge fossil record like *cetaceans* could benefit from our approach. This dataset is characterized by a rather small number of extant species and fossils with morphological data available (respectively ρ-sampled and ψ-sampled species), but includes a huge number of fossils without morphological data (ω-sampled species) (Morlon et al., 2011, Barido-Sottani et al., 2019). For the cetaceans as well as many other clades, it will be of great interest to compute diversity estimates under the modelling framework presented here (assuming ρ≠0,ω≠0,r=0). Ultimately, all ω-samples could be taken into account to inform the tree and diversity estimates.

In the context of epidemiology, typically, the genetic sequences of the pathogen are only available for a fraction of the infected individuals. These correspond to ψ-samples, while other sampled infected individuals correspond to ω-samples. Further developing our approach in a Bayesian framework, both the genetic sequences and the record of occurrence could be jointly used to estimate the underlying transmission tree and prevalence of the disease through time. Depending on the cost of sequencing and the ability of numerical methods to handle some critical amount of both genetic sequences and number of occurrences, optimal sampling procedure could be investigated, to make the most of both types of data.

Finally, while improving on current methods, these two Algorithms 1 and 2 still only provide approximations of, respectively, $L_{t}$ and $M_{t}$, that critically rely on the truncation parameter of the state space *N*. Increasing *N* leads to a more accurate approximation, while increasing the runtime of the method. If the probability mass of the number of hidden individuals is non-negligible above *N*, both algorithms will lead to very poor approximations of $L_{t}$ and $M_{t}$. This value should thus be carefully chosen in empirical applications, depending on what is expected with the data at hand. We point out that the behaviour of these algorithms strongly relies on the runtime and accurracy of the matrix exponentiation steps. Numerous matrix exponentiation methods have been proposed in the literature (Moler and Van Loan, 2003). In our current implementation, we rely on a recent matrix exponentiation method already implemented in *scipy* (Al-Mohy and Higham, 2010). Future avenues towards improving this specific step could focus on new theoretical results adapted to tridiagonal matrices (Smith and Shahrezaei, 2015) or alternatively try to adapt Laplace transform approximations derived in Crawford et al. (2014), who present theoretical results bounding the errors made in their approximation.

### 6.4 Future extensions

Our proposed modelling framework lends itself well for various biologically realistic extensions to allow closer fit to empirical data in a variety of situations.

The first extension that we envision is to relax the assumption of rate homogeneity and instead work with time-varying rates. This has already been considered in different studies relying on birth-death processes, either with exponentially varying functions (Morlon et al., 2011) or with piecewise constant rates (a model dubbed as *skyline birth-death process*, see Stadler et al., 2013, Gavryushkina et al., 2016). As all our results can be straightforwardly adapted to such a framework, this would not require much theoretical work. However, the challenge would be to do so without overfitting the data.

Another popular extension that has been described in the literature on birth-death processes for phylodynamics is to consider multi-type birth-death processes (Maddison et al., 2007). Each individual is assigned a type, which impacts its propensity to give birth to other types. All sampling-related parameters can also be considered type-dependent. The main challenge here boils down to dealing with an increase of dimensionality, because we would be interested in the joint distribution of all subpopulation sizes. This extension is particularly interesting for epidemiological applications, when different populations of infected individuals, clustered according to some characteristic (e.g. patient behaviour or geography) might have very different dynamics (Stadler and Bonhoeffer, 2013).

Finally, we are very hopeful that this piece of work could be applied as well to density-dependent birth-death processes, also known as *logistic birth-death models*. Indeed, very similar ideas to the breadth-first forward and backward traversals as applied in Algorithms 1’ and 2’ appear in the context of logistic birth-death models (Etienne et al., 2012, Leventhal et al., 2013, Laudanno et al., 2020). Preliminary results obtained by adapting our numerical algorithms to this framework are very encouraging, and we are currently in the process of deriving as much analytical results as we can to speed up the method. We are hoping to present this in a subsequent paper.

### 6.5 Conclusion

This manuscript presents a way to efficiently compute the distribution of the past population size in a linear birth-death process, conditioned on the observation of a reconstructed phylogenetic tree and a record of occurrences through time. Such data are very common in macroevolution where the reconstructed phylogenetic tree of extant species is available together with occurrences from the fossil record. In epidemiology, pathogen genetic sequencing data and case count data are a common data source. Our method thus promises to allow efficient quantification of past population sizes, representing past biodiversity or past prevalence, from these rich datasets.

We believe that this method also paves the way for the consideration of more complex and more realistic demographic scenarios, assuming either time-dependent (Morlon et al., 2011, Stadler et al., 2013, Gavryushkina et al., 2016) or density-dependent parameters (Etienne et al., 2012, Leventhal et al., 2013), potentially catering for populations with multiple demographic categories/types (Maddison et al., 2007, Stadler and Bonhoeffer, 2013, Freyman and Höhna, 2018). It is our hope that this manuscript will foster important research advances for unravelling demographic histories in epidemiology, macroevolution, and any other fields where birth-death processes form a relevant model framework.

## Declaration of Competing Interest

The authors declare that they have no known competing financial interests or personal relationships that could have appeared to influence the work reported in this paper.

## CRediT authorship contribution statement

**Marc Manceau:** Conceptualization, Methodology, Software, Validation, Formal analysis, Investigation, Writing - original draft. **Ankit Gupta:** Validation, Investigation, Writing - original draft, Visualization. **Timothy Vaughan:** Validation, Investigation, Writing - review & editing, Supervision. **Tanja Stadler:** Conceptualization, Resources, Writing - review & editing, Supervision, Funding acquisition.

## Appendix A Solving well-known ODEs

### A.1 The extinction probability

We first deal with Eq. (2.1) governing $u_{t}$, and start by studying the polynomial $\lambda x^{2}-\gamma x+\mu$.

This polynomial has discriminant $\Delta =\gamma^{2}-4\lambda \mu >{\left(\lambda +\mu \right)}^{2}-4\lambda \mu ⩾{\left(\lambda -\mu \right)}^{2}⩾0$. Note that the first inequality holds in the case we are interested in because we can assume that ψ+ω>0. When this is not the case, one needs to consider that λ≠μ. Roots are

$$x_{1}=\frac{\gamma -\sqrt{\Delta }}{2\lambda }\;\mathrm{and}\;x_{2}=\frac{\gamma +\sqrt{\Delta }}{2\lambda }\text{.}$$

Moreover, we know that both roots are positive because $\Delta <\gamma^{2}\Rightarrow \sqrt{\Delta }<\gamma \Rightarrow x_{1}>0$.

On an interval including zero and where the polynomial remains positive (as $\left(-\infty ,x_{1}\right)$ for example), we can write,

$$\begin{array}{cc} & \frac{\mathrm{du}}{\lambda u^{2}-\gamma u+\mu }=\mathrm{dt}\\ \Leftrightarrow & \frac{\mathrm{du}}{\left(x_{1}-u)(x_{2}-u\right)}=\lambda \mathrm{dt}\\ \Leftrightarrow & \frac{1}{x_{2}-x_{1}}\left(\frac{1}{x_{1}-u}-\frac{1}{x_{2}-u}\right)\mathrm{du}=\lambda \mathrm{dt}\\ \Leftrightarrow & \left(\frac{1}{x_{1}-u}-\frac{1}{x_{2}-u}\right)\mathrm{du}=\sqrt{\Delta }\mathrm{dt}\text{.} \end{array}$$

Integrating both sides between time 0 and *t*, we get

$$\begin{array}{cc} & \frac{x_{2}-u_{t}}{x_{1}-u_{t}}=\frac{x_{2}-z}{x_{1}-z}e^{\sqrt{\Delta }t}\\ \Leftrightarrow & x_{2}(x_{1}-z)e^{-\sqrt{\Delta }t}-u_{t}(x_{1}-z)e^{-\sqrt{\Delta }t}=x_{1}(x_{2}-z)-u_{t}(x_{2}-z)\\ \Leftrightarrow & u_{t}\left((x_{2}-z)-(x_{1}-z)e^{-\sqrt{\Delta }t}\right)=x_{1}(x_{2}-z)-x_{2}(x_{1}-z)e^{-\sqrt{\Delta }t}\\ \Leftrightarrow & u_{t}=\frac{x_{1}(x_{2}-z)-x_{2}(x_{1}-z)e^{-\sqrt{\Delta }t}}{(x_{2}-z)-(x_{1}-z)e^{-\sqrt{\Delta }t}}\text{.} \end{array}$$

This is the result stated in Eq. (2.2). Note that this quantity is called $p_{0}(t)$ in Stadler (2010), or E(t) in Maddison et al. (2007).

### A.2 Probability to leave only one sampled descendent

We aim here to integrate a slight variation of Eq. (2.3) governing $p_{t}$ when k=1. The equation we are interested in is,

(A.1) $$\frac{{\mathrm{dW}}_{s}}{\mathrm{ds}}=(2\lambda u(s,z)-\gamma ){\mathrm{kW}}_{s}$$

$$\begin{array}{cc} \frac{{\mathrm{dW}}_{s}}{W_{s}}= & \left(2\lambda k\frac{x_{1}(x_{2}-z)-x_{2}(x_{1}-z)e^{-\sqrt{\Delta }s}}{(x_{2}-z)-(x_{1}-z)e^{-\sqrt{\Delta }s}}-\gamma k\right)\mathrm{ds}\\ = & \left(\frac{2\lambda {\mathrm{kx}}_{1}}{\sqrt{\Delta }}\frac{\sqrt{\Delta }(x_{2}-z)e^{\sqrt{\Delta }s}}{\left(x_{2}-z)e^{\sqrt{\Delta }s}-(x_{1}-z\right)}-\frac{2\lambda {\mathrm{kx}}_{2}}{\sqrt{\Delta }}\frac{(x_{1}-z)\sqrt{\Delta }e^{-\sqrt{\Delta }s}}{(x_{2}-z)-(x_{1}-z)e^{-\sqrt{\Delta }s}}-\gamma k\right)\mathrm{ds}\text{.} \end{array}$$

All these three terms can be integrated visually between some time $t_{h}$ and *t*, leading to,

$$\begin{array}{cc} \ln\frac{W_{t}}{W_{t_{h}}}= & \frac{2\lambda {\mathrm{kx}}_{1}}{\sqrt{\Delta }}{\left[\ln\left(\left(x_{2}-z)e^{\sqrt{\Delta }s}-(x_{1}-z\right)\right)\right]}_{t_{h}}^{t}-\frac{2\lambda {\mathrm{kx}}_{2}}{\sqrt{\Delta }}{\left[\ln\left((x_{2}-z)-(x_{1}-z)e^{-\sqrt{\Delta }s}\right)\right]}_{t_{h}}^{t}-\gamma k(t-t_{h})\\ = & \frac{2\lambda {\mathrm{kx}}_{1}}{\sqrt{\Delta }}\ln\frac{\left(x_{2}-z)e^{\sqrt{\Delta }t}-(x_{1}-z\right)}{\left(x_{2}-z)e^{\sqrt{\Delta }t_{h}}-(x_{1}-z\right)}-\frac{2\lambda {\mathrm{kx}}_{2}}{\sqrt{\Delta }}\ln\frac{(x_{2}-z)-(x_{1}-z)e^{-\sqrt{\Delta }t}}{(x_{2}-z)-(x_{1}-z)e^{-\sqrt{\Delta }t_{h}}}-\gamma k(t-t_{h})\\ = & -\frac{2\lambda k(x_{2}-x_{1})}{\sqrt{\Delta }}\ln\frac{(x_{2}-z)-(x_{1}-z)e^{-\sqrt{\Delta }t}}{(x_{2}-z)-(x_{1}-z)e^{-\sqrt{\Delta }t_{h}}}-\gamma k(t-t_{h})+2\lambda {\mathrm{kx}}_{1}(t-t_{h})\\ = & -2k\ln\frac{(x_{2}-z)-(x_{1}-z)e^{-\sqrt{\Delta }t}}{(x_{2}-z)-(x_{1}-z)e^{-\sqrt{\Delta }t_{h}}}-k\sqrt{\Delta }(t-t_{h})\text{.} \end{array}$$

Leading to the final expression below

(A.2) $$W_{t}=W_{t_{h}}{\left(\frac{(x_{2}-z)-(x_{1}-z)e^{-\sqrt{\Delta }t}}{(x_{2}-z)-(x_{1}-z)e^{-\sqrt{\Delta }t_{h}}}\right)}^{-2k}e^{-k\sqrt{\Delta }(t-t_{h})}\text{.}$$

Note that the case $k=1,t_{h}=0$ and $W_{0}=1-z$ corresponds to the probability $p_{t}$ given as Eq. (2.4),

$$p(t,z)=(1-z)\frac{\Delta }{\lambda^{2}}{\left((x_{2}-z)-(x_{1}-z)e^{-\sqrt{\Delta }t}\right)}^{-2}e^{-\sqrt{\Delta }t}\text{.}$$

while the general case can be expressed using function *p* as

$$W_{t}=W_{t_{h}}{\left(\frac{p(t,z)}{p(t_{h},z)}\right)}^{k}\text{.}$$

### A.3 A few useful properties

Solutions u(t,z) and p(t,z) to ODEs (2.1), (2.3) satisfy two properties relying on the semi-group property of solutions of ODEs, namely,

(A.3) $$u(t_{2}-t_{1},u(t_{1},z))=u(t_{2},z)$$

(A.4) $$p(t_{2}-t_{1},u(t_{1},z))=\frac{p(t_{2},z)p(0,u(t_{1},z))}{p(t_{1},z)}=\frac{p(t_{2},z)(1-u(t_{1},z))}{p(t_{1},z)}\text{.}$$

These two properties are useful in many calculations throughout this document, e.g.

• Solving the main PDE in Appendix C requires inverting *u*, using the first property with,
  $$\begin{array}{cc} & z=u(t-t_{h},z_{0})\\ \Leftrightarrow & u(t_{h}-t,z)=u(t_{h}-t,u(t-t_{h},z_{0}))\\ \Leftrightarrow & u(t_{h}-t,z)=u(0,z_{0})\\ \Leftrightarrow & z_{0}=u(t_{h}-t,z)\text{.} \end{array}$$
• The same Appendix section requires also composing function *p* and *u*, using
  $$\frac{p(t-t_{h},u(t_{h}-t,z))}{p(0,u(t_{h}-t,z)}=\frac{p(0,z)}{p(t_{h}-t,z)}\text{.}$$
• In the proof of Proposition 4.3, we switch to the notation R(t,z)=p(t,z)/(1-z) and again compose *R* and *u* in the same way,
  $$\begin{array}{cc} & \frac{p(t_{j}-t,u(t_{h}-t,z))}{p(0,u(t_{h}-t,z))}=\frac{p(t_{j}-t,z)}{p(t_{h}-t,z)}\\ \Leftrightarrow & \frac{p(t_{j}-t,u(t_{h}-t,z))}{1-u(t_{h}-t,z)}=\frac{p(t_{j}-t,z)}{p(t_{h}-t,z)}\\ \Leftrightarrow & R(t_{j}-t_{h},u(t_{h}-t,z))=\frac{R(t_{j}-t,z)}{R(t_{h}-t,z)}\text{.} \end{array}$$

## Appendix B Link with previous work by Gupta et al. (2019)

We aim here at providing details to link this work with results previously derived by Gupta et al. (2019), allowing efficient computation of $L_{t}^{\left(i\right)}$ in the special case r=1.

To do so, we define the distinguishable version of the probability $L_{t}^{\left(i\right)}$ as

(B.1) $${\overset{‾}{L}}_{t}^{\left(i\right)}=\frac{(k+i)!}{i!}L_{t}^{\left(i\right)}\text{.}$$

We now derive the ODE for ${\overset{‾}{L}}_{t}^{\left(i\right)}$. Multiplying both sides of (3.2) by $\frac{(k+i)!}{i!}$we obtain

(B.2) $$\begin{array}{cc} {\overset{˙}{\overset{‾}{L}}}_{t}^{\left(i\right)}= & -\gamma (k+i)\frac{(k+i)!}{i!}L_{t}^{\left(i\right)}+\lambda (2k+i)\frac{(k+i)!}{i!}L_{t}^{\left(i+1\right)}+\mu i\frac{(k+i)!}{i!}L_{t}^{\left(i-1\right)}\\ & =-\gamma (k+i)\frac{(k+i)!}{i!}L_{t}^{\left(i\right)}+\lambda (k+i)\left[\frac{\left(2k+i)(i+1\right)}{\left(k+i+1)(k+i\right)}\right]\frac{(k+i+1)!}{(i+1)!}L_{t}^{\left(i+1\right)}+\mu (k+i)\frac{(k+i-1)!}{(i-1)!}L_{t}^{\left(i-1\right)}\\ & =-\gamma (k+i){\overset{‾}{L}}_{t}^{\left(i\right)}+\lambda (k+i)\phi_{i,k}{\overset{‾}{L}}_{t}^{\left(i+1\right)}+\mu (k+i){\overset{‾}{L}}_{t}^{\left(i-1\right)}, \end{array}$$

where

$$\phi_{i,k}=\frac{\left(2k+i)(i+1\right)}{\left(k+i+1)(k+i\right)}=1-\frac{k(k-1)}{\left(k+i+1)(k+i\right)}$$

is the probability that a coalescing pair of randomly chosen lineages (from (k+i+1) total lineages) does not consist of two sampled lineages. This shows that ${\overset{‾}{L}}_{t}^{\left(i\right)}$ satisfies the ODE (B.2) across any epoch. One can see that at punctual events the transition conditions (3.3), (3.8) hold for ${\overset{‾}{L}}_{t}^{\left(i\right)}$ for ψ-sampling and branching events respectively. Moreover at ω-sampling events the transition condition (3.6) transforms to

$${\overset{‾}{L}}_{t^{+}}^{\left(i\right)}=\omega (k+i){\overset{‾}{L}}_{t^{-}}^{\left(i-1\right)}\text{.}$$

With these transition conditions and initial condition ${\overset{‾}{L}}_{0}^{\left(i\right)}=\frac{(k_{0}+i)!}{i!}L_{0}^{\left(i\right)}=\frac{(k_{0}+i)!}{i!}{\left(1-\rho \right)}^{k_{0}}\rho^{i}$, the ODE (B.2) was solved explicitly in Gupta et al. (2019) and the solution is of the form

$${\overset{‾}{L}}_{t^{+}}^{\left(i\right)}=\overset{q}{\underset{\ell =0}{\sum }}\frac{(k+i)!}{(i-\ell )!}u_{t}^{i-\ell }W_{t}^{\left(\ell \right)}$$

where *q* is the number of ω-sampling events in the time-interval [0,t) and the (q+1)-dimensional time-varying vector $W_{t}=(W_{t}^{\left(0\right)},\ldots ,W_{t}^{\left(q\right)})$ can be analytically computed following the approach in Gupta et al. (2019). Therefore from (B.1) we state Proposition 3.2.

## Appendix C Solving the main PDE

We aim now at finding an analytical solution for the following PDE, driving the evolution of M^ across a given epoch $\left(t_{h-1},t_{h}\right)$, on which the number of observed lineages remains constant and equal to *k*,

M^(th,z)=F(z)∂tM^+(λz2-γz+μ)∂zM^+k(2λz-γ)M^=0.

### C.1 Principle of the method of characteristics

This problem can be solved by the method of characteristics. We suppose that we can write M^(t,z)=M^(t(s),z(s)) where functions *t* and *z* satisfy the ODEs,

$$\begin{array}{cc} \frac{\mathrm{dz}}{\mathrm{ds}}= & \lambda z^{2}-\gamma z+\mu \\ \frac{\mathrm{dt}}{\mathrm{ds}}= & 1\text{.} \end{array}$$

This way, the function g(s)=M^(t(s),z(s)) satisfies another ODE, that we will have to solve,

dgds=dzds∂zM^+dtds∂tM^=(λz2-γz+μ)∂zM^+∂tM^=-k(2λz-γ)M^⇔dgds+k(2λz-γ)g=0.

### C.2 Step 1, solve for t(s),z(s) and g(s)

We start by integrating t(s). We moreover fix that $t(0)=t_{h}$, thus leading to $t(s)=t_{h}+s$.

We now turn to *z*, and notice that it satisfies previously studied ODE (2.1). Integrating between 0 and *s* leads to,

$$z(s)=u(s,z_{0})=\frac{x_{1}(x_{2}-z_{0})-x_{2}(x_{1}-z_{0})e^{-\sqrt{\Delta }t}}{(x_{2}-z_{0})-(x_{1}-z_{0})e^{-\sqrt{\Delta }t}}\text{.}$$

Last, *g* satisfies an ODE very similar to (A.1). Taking care of the minus sign, it leads to the following result,

(C.1) $$g_{s}=g_{0}{\left(\frac{1}{R(s,z_{0})}\right)}^{k}\text{.}$$

### C.3 Step 2, express M^ back as a function of t,z

We want to express our two unknown quantities *s* and $z_{0}$ as functions of *t* and *z*.

On a first hand, we get easily $s=t-t_{h}$. We moreover can solve for $z_{0}$ in the following equation, remembering the semi-group property of *u*,

$$z=u(t-t_{h},z_{0})\;\Leftrightarrow \;z_{0}=u(t_{h}-t,z)\text{.}$$

Substituting these into the previous expression (C.1) of $g_{s}$ then leads to,

M^(t,z)=Fu(th-t,z)R(t-th,u(th-t,z))-k=Fu(th-t,z)R(th-t,z)k.

where the first to second equality relies on a property exposed in A.3. This gives us the final formula which is stated in Proposition 4.1.

## Appendix D Some useful algebra

This section of the Appendix pools together all bits of algebra that are not really digestible, but are used in the main text.

### D.1 Deivative of M^

We first modify a bit the expression of the generating function,

M^(th-t,z)=R(th-t,z)k∑l=0NM∼th(l)u(th-t,z)l=Δλ2e-Δtk∑l=0NM∼th(l)x1(x2-z)-x2(x1-z)e-Δtl(x2-z)-(x1-z)e-Δt-(2k+l).

Applying Leibniz’s derivation rule to the product, we get,

∂ziM^(th-t,z)z=0=Δλ2e-Δtk∑l=0NM∼th(l)∑α=0iiα∂zαx1(x2-z)-x2(x1-z)e-Δtlz=0

(D.1) $${\left(\partial_{z}^{i-\alpha }{\left((x_{2}-z)-(x_{1}-z)e^{-\sqrt{\Delta }t}\right)}^{-(2k+l)}\right)}_{z=0}\text{.}$$

The first of the two derivatives in the sum can be computed as,

$$\begin{array}{cc} \partial_{z}{\left(x_{1}(x_{2}-z)-x_{2}(x_{1}-z)e^{-\sqrt{\Delta }t}\right)}^{l}= & l\left(-x_{1}+x_{2}e^{-\sqrt{\Delta }t}\right){\left(x_{1}(x_{2}-z)-x_{2}(x_{1}-z)e^{-\sqrt{\Delta }t}\right)}^{l-1}1_{l⩾1}\\ \partial_{z}^{2}{\left(x_{1}(x_{2}-z)-x_{2}(x_{1}-z)e^{-\sqrt{\Delta }t}\right)}^{l}= & l(l-1){\left(-x_{1}+x_{2}e^{-\sqrt{\Delta }t}\right)}^{2}{\left(x_{1}(x_{2}-z)-x_{2}(x_{1}-z)e^{-\sqrt{\Delta }t}\right)}^{l-2}1_{l⩾2}\\ & \vdots \\ \partial_{z}^{\alpha }{\left(x_{1}(x_{2}-z)-x_{2}(x_{1}-z)e^{-\sqrt{\Delta }t}\right)}^{l}= & \frac{l!}{(l-\alpha )!}{\left(-x_{1}+x_{2}e^{-\sqrt{\Delta }t}\right)}^{\alpha }{\left(x_{1}(x_{2}-z)-x_{2}(x_{1}-z)e^{-\sqrt{\Delta }t}\right)}^{l-\alpha }1_{l⩾\alpha }\text{.} \end{array}$$

While the second gives us,

$$\begin{array}{cc} \partial_{z}{\left((x_{2}-z)-(x_{1}-z)e^{-\sqrt{\Delta }t}\right)}^{-(2k+l)}= & (2k+l)\left(1-e^{-\sqrt{\Delta }t}\right){\left((x_{2}-z)-(x_{1}-z)e^{-\sqrt{\Delta }t}\right)}^{-(2k+l+1)}\\ \partial_{z}^{2}{\left((x_{2}-z)-(x_{1}-z)e^{-\sqrt{\Delta }t}\right)}^{-(2k+l)}= & (2k+l)(2k+l+1){\left(1-e^{-\sqrt{\Delta }t}\right)}^{2}{\left((x_{2}-z)-(x_{1}-z)e^{-\sqrt{\Delta }t}\right)}^{-(2k+l+2)}\\ & \;\;\;\vdots \\ \partial_{z}^{i-\alpha }{\left((x_{2}-z)-(x_{1}-z)e^{-\sqrt{\Delta }t}\right)}^{-(2k+l)}= & \left(\overset{i-\alpha -1}{\underset{m=0}{\prod }}(2k+l+m)\right){\left(1-e^{-\sqrt{\Delta }t}\right)}^{i-\alpha }\\ & {\left((x_{2}-z)-(x_{1}-z)e^{-\sqrt{\Delta }t}\right)}^{-(2k+l+i-\alpha )}\text{.} \end{array}$$

Applying these derivatives in z=0 in Eq. (D.1) yields,

∂ziM^(th-t,z)z=0=Δλ2e-Δtk∑α=0i∑l=αNM∼th(l)iαl!(l-α)!∏m=0i-α-1(2k+l+m)-x1+x2e-Δtαx1x2l-α1-e-Δtl+i-2αx2-x1e-Δt-(2k+l+i-α)

which is the expression provided in Proposition (4.2).

### D.2 Derivatives of M^ when ω=0

We wish here to derive the ∂ziM^(t,z) where function M^ is as given in Proposition 4.3, i.e.

M^(t,z)=λxψv+w+yrw(1-r)v+yR(tor-t,z)∏tj∈XR(tj-t,z)∏tj∈WR(tj-t,z)-1∏tj∈Yu(tj-t,z)R(tj-t,z)-1.

We take for simplicity the derivative of the logarithm of M^ and express the derivatives of M^ using these and Leibniz’s formula,

(D.2) ∂zM^=M^∂z(lnM^)∂z2M^=∂zM^∂z(lnM^)+M^∂z2(lnM^)∂z3M^=∂z2M^∂z(lnM^)+2∂zM^∂z2(lnM^)+M^∂z3(lnM^)⋮∂ziM^=∑α=1ii-1α-1∂zi-αM^∂zα(lnM^).

In order to compute the derivatives of lnM^, one needs to get the derivatives of lnR(t,z) and lnu(t,z). We have

$$\begin{array}{cc} \ln R(t,z)= & -2\ln\left((x_{2}-z)-(x_{1}-z)e^{-\sqrt{\Delta }t}\right)+\ln\frac{\Delta }{\lambda^{2}}-\sqrt{\Delta }t\\ \partial_{z}\ln R(t,z)= & 2\left(1-e^{-\sqrt{\Delta }t}\right){\left((x_{2}-z)-(x_{1}-z)e^{-\sqrt{\Delta }t}\right)}^{-1}\\ \partial_{z}^{2}\ln R(t,z)= & 2{\left(1-e^{-\sqrt{\Delta }t}\right)}^{2}{\left((x_{2}-z)-(x_{1}-z)e^{-\sqrt{\Delta }t}\right)}^{-2}\\ \partial_{z}^{3}\ln R(t,z)= & 4{\left(1-e^{-\sqrt{\Delta }t}\right)}^{3}{\left((x_{2}-z)-(x_{1}-z)e^{-\sqrt{\Delta }t}\right)}^{-3}\\ & \;\;\vdots \\ \partial_{z}^{\alpha }\ln R(t,z)= & 2(\alpha -1)!{\left(1-e^{-\sqrt{\Delta }t}\right)}^{\alpha }{\left((x_{2}-z)-(x_{1}-z)e^{-\sqrt{\Delta }t}\right)}^{-\alpha }\text{.} \end{array}$$

Finally taking the function in z=0 leads to

(D.3) $$\partial_{z}^{\alpha }\ln R(t,0)=2(\alpha -1)!a_{t}^{\alpha }$$

(D.4) $$\text{where we defined}\;a_{t}≔\left(1-e^{-\sqrt{\Delta }t}\right){\left(x_{2}-x_{1}e^{-\sqrt{\Delta }t}\right)}^{-1}\text{.}$$

In the same way we get,

$$\begin{array}{cc} \ln u(t,z)= & \ln\left(x_{1}(x_{2}-z)-x_{2}(x_{1}-z)e^{-\sqrt{\Delta }t}\right)-\ln\left((x_{2}-z)-(x_{1}-z)e^{-\sqrt{\Delta }t}\right)\\ \partial_{z}\ln u(t,z)= & -\left(x_{1}-x_{2}e^{-\sqrt{\Delta }t}\right){\left(x_{1}(x_{2}-z)-x_{2}(x_{1}-z)e^{-\sqrt{\Delta }t}\right)}^{-1}\\ & +\left(1-e^{-\sqrt{\Delta }t}\right){\left((x_{2}-z)-(x_{1}-z)e^{-\sqrt{\Delta }t}\right)}^{-1}\\ \partial_{z}^{2}\ln u(t,z)= & -{\left(x_{1}-x_{2}e^{-\sqrt{\Delta }t}\right)}^{2}{\left(x_{1}(x_{2}-z)-x_{2}(x_{1}-z)e^{-\sqrt{\Delta }t}\right)}^{-2}\\ & +{\left(1-e^{-\sqrt{\Delta }t}\right)}^{2}{\left((x_{2}-z)-(x_{1}-z)e^{-\sqrt{\Delta }t}\right)}^{-2}\\ \partial_{z}^{3}\ln u(t,z)= & -2{\left(x_{1}-x_{2}e^{-\sqrt{\Delta }t}\right)}^{3}{\left(x_{1}(x_{2}-z)-x_{2}(x_{1}-z)e^{-\sqrt{\Delta }t}\right)}^{-3}\\ & +2{\left(1-e^{-\sqrt{\Delta }t}\right)}^{3}{\left((x_{2}-z)-(x_{1}-z)e^{-\sqrt{\Delta }t}\right)}^{-3}\\ & \;\;\vdots \\ \partial_{z}^{\alpha }\ln u(t,z)= & (\alpha -1)!\left[-{\left(x_{1}-x_{2}e^{-\sqrt{\Delta }t}\right)}^{\alpha }{\left(x_{1}(x_{2}-z)-x_{2}(x_{1}-z)e^{-\sqrt{\Delta }t}\right)}^{-\alpha }\right)\\ & \left(+{\left(1-e^{-\sqrt{\Delta }t}\right)}^{\alpha }{\left((x_{2}-z)-(x_{1}-z)e^{-\sqrt{\Delta }t}\right)}^{-\alpha }\right]\text{.} \end{array}$$

Here also, we are interested in the function in z=0,

(D.5) $$\partial_{z}^{\alpha }\ln u(t,0)=(\alpha -1)!(a_{t}^{\alpha }-b_{t}^{\alpha })$$

(D.6) $$\text{where we defined}\;b_{t}≔\left(x_{1}-x_{2}e^{-\sqrt{\Delta }t}\right){\left(x_{1}x_{2}-x_{2}x_{1}e^{-\sqrt{\Delta }t}\right)}^{-1}\text{.}$$

Last ingredient needed to write the derivative of lnM^, we get,

(D.7) $$\begin{array}{cc} {\left(\partial_{z}^{\alpha }\ln\left(u(t,z)R{\left(t,z\right)}^{-1}\right)\right)}_{z=0}= & {\left(\partial_{z}^{\alpha }\ln u(t,z)\right)}_{z=0}-{\left(\partial_{z}^{\alpha }\ln R(t,z)\right)}_{z=0}\\ = & -(\alpha -1)!(a_{t}^{\alpha }+b_{t}^{\alpha })\text{.} \end{array}$$

Finally, using Eq. (D.1)one can compute

∂zα(lnM^(t,z))z=0=(α-1)!C(α)where we definedC(α)≔2ator-tα+2∑tj∈Xatj-tα-2∑tj∈Watj-tα-∑tj∈Y(atj-tα+btj-tα).

Plugging this into Eq. D.2 and noting that ∂ziM^(t,z)z=0=i!Mt(i), we get

$$M_{t}^{\left(i\right)}=\overset{i}{\underset{\alpha =1}{\sum }}\left(\begin{array}{c} i-1\\ \alpha -1 \end{array}\right)\frac{(i-\alpha )!(\alpha -1)!}{i!}M_{t}^{\left(i-\alpha \right)}C^{\left(\alpha \right)}=\frac{1}{i}\overset{i}{\underset{\alpha =1}{\sum }}M_{t}^{\left(i-\alpha \right)}C^{\left(\alpha \right)}$$

which is the result stated in Corollary 4.3.1.

## Appendix E Inductions across the epochs

### E.1 Proof of Proposition 3.1

We prove the proposition by induction across the epochs.

If we observe only the first epoch and the $k_{0}$ leaves at present, then we get at any time *t* across the first epoch $(0,t_{1}),\;L_{t}^{\left(i\right)}=\rho^{k_{0}}{\left(1-\rho \right)}^{i}=u_{t}^{i}p_{t}^{k_{0}}$, which satisfies Proposition 3.1.

Suppose we observed so far – i.e. on $\left(0,t_{h+1}\right)$ – *v* sampled ancestors, *w* removed leaves at times $t_{j}\in W$, *x* branching events at times $t_{j}\in X$, *y* non-removed leaves at times $t_{j}\in Y$. And suppose that Proposition 3.1 is verified across epoch $\left(t_{h},t_{h+1}\right)$. Let us have a look at what happen across epoch $\left(t_{h+1},t_{h+2}\right)$.

The observed punctual event $t_{h+1}$ can either be,

1. a removed ancestral leaf. Update (3.10) then applies. Subsequently, the number of sampled lineages increases by one and formula (3.9) applies on the next epoch, leading to
  $$\begin{array}{cc} L_{t}^{\left(i\right)}= & u_{t}^{i}W_{t}\\ \mathrm{where}\;W_{t}= & \lambda^{x}\psi^{v+(w+1)+y}{\left(1-r\right)}^{v+y}r^{w+1}p_{t_{h+1}}^{k}{\left(\frac{p_{t}}{p_{t_{h+1}}}\right)}^{k+1}\underset{t_{j}\in X}{\prod }p_{t_{j}}\underset{t_{j}\in Y}{\prod }u_{t_{j}}p_{t_{j}}^{-1}\underset{t_{j}\in W}{\prod }p_{t_{j}}^{-1}\\ = & \lambda^{x}\psi^{v+(w+1)+y}{\left(1-r\right)}^{v+y}r^{w+1}p_{t}^{k}\underset{t_{j}\in X}{\prod }p_{t_{j}}\underset{t_{j}\in Y}{\prod }u_{t_{j}}p_{t_{j}}^{-1}\underset{t_{j}\in W\cup \{t_{h+1}\}}{\prod }p_{t_{j}}^{-1}\text{.} \end{array}$$
2. a non-removed ancestral leaf. Update (3.11) then applies. Subsequently, the number of sampled lineages increases by one and formula (3.9) applies on the next epoch, leading to
  $$\begin{array}{cc} L_{t}^{\left(i\right)}= & u_{t}^{i}W_{t}\\ \mathrm{where}\;W_{t}= & \lambda^{x}\psi^{v+w+(y+1)}{\left(1-r\right)}^{v+(y+1)}r^{w}p_{t_{h+1}}^{k}u_{t_{h+1}}{\left(\frac{p_{t}}{p_{t_{h+1}}}\right)}^{k+1}\underset{t_{j}\in X}{\prod }p_{t_{j}}\underset{t_{j}\in Y}{\prod }u_{t_{j}}p_{t_{j}}^{-1}\underset{t_{j}\in W}{\prod }p_{t_{j}}^{-1}\\ = & \lambda^{x}\psi^{v+w+(y+1)}{\left(1-r\right)}^{v+(y+1)}r^{w}p_{t}^{k+1}\underset{t_{j}\in X}{\prod }p_{t_{j}}\underset{t_{j}\in Y\cup \{t_{h+1}\}}{\prod }u_{t_{j}}p_{t_{j}}^{-1}\underset{t_{j}\in W}{\prod }p_{t_{j}}^{-1}\text{.} \end{array}$$
3. a non-removed sampled ancestor along a branch. Update (3.12) then applies. The number of sampled lineages does not changes, and formula (3.9) applies on the next epoch, leading to
  $$\begin{array}{cc} L_{t}^{\left(i\right)}= & u_{t}^{i}W_{t}\\ \mathrm{where}\;W_{t}= & \lambda^{x}\psi^{(v+1)+w+y}{\left(1-r\right)}^{(v+1)+y}r^{w}p_{t_{h+1}}^{k}{\left(\frac{p_{t}}{p_{t_{h+1}}}\right)}^{k}\underset{t_{j}\in X}{\prod }p_{t_{j}}\underset{t_{j}\in Y}{\prod }u_{t_{j}}p_{t_{j}}^{-1}\underset{t_{j}\in W}{\prod }p_{t_{j}}^{-1}\\ = & \lambda^{x}\psi^{v+w+y+1}{\left(1-r\right)}^{v+y+1}r^{w}p_{t}^{k}\underset{t_{j}\in X}{\prod }p_{t_{j}}\underset{t_{j}\in Y}{\prod }u_{t_{j}}p_{t_{j}}^{-1}\underset{t_{j}\in W}{\prod }p_{t_{j}}^{-1}\text{.} \end{array}$$
4. a branching event between two sampled lineages. Update (3.13) then applies. The number of sampled lineages decreases by one, and formula (3.9) applies on the next epoch, leading to
  $$\begin{array}{cc} L_{t}^{\left(i\right)}= & u_{t}^{i}W_{t}\\ \mathrm{where}\;W_{t}= & \lambda^{x+1}\psi^{v+w+y}{\left(1-r\right)}^{v+y}r^{w}p_{t_{h+1}}^{k}{\left(\frac{p_{t}}{p_{t_{h+1}}}\right)}^{k-1}\underset{t_{j}\in X}{\prod }p_{t_{j}}\underset{t_{j}\in Y}{\prod }u_{t_{j}}p_{t_{j}}^{-1}\underset{t_{j}\in W}{\prod }p_{t_{j}}^{-1}\\ = & \lambda^{x+1}\psi^{v+w+y}{\left(1-r\right)}^{v+y}r^{w}p_{t}^{k-1}\underset{t_{j}\in X\cup \{t_{h+1}\}}{\prod }p_{t_{j}}\underset{t_{j}\in Y}{\prod }u_{t_{j}}p_{t_{j}}^{-1}\underset{t_{j}\in W}{\prod }p_{t_{j}}^{-1}\text{.} \end{array}$$

In all four cases, Proposition 3.1 is satisfied across epoch $\left(t_{h+1},t_{h+2}\right)$.

### E.2 Proof of Proposition 4.3

This Proposition is also proven by induction across the epochs.

We start at $t_{\mathrm{or}}=t_{n}$ with k=1 lineage. Across epoch $\left(t_{n-1},t_{n}\right)$, applying Proposition 4.1 with F(z)=1 and k=1, we get M^(t,z)=R(tor-t,z), which verifies Proposition 4.3.

Suppose now that Proposition 4.3 is verified across epoch $\left(t_{h},t_{h+1}\right)$ and that we observed, on $\left(t_{h},t_{\mathrm{or}}\right)$, *v* sampled ancestors, *w* removed leaves at times $t_{j}\in W$, *x* branching events at times $t_{j}\in X$, *y* non-removed leaves at times $t_{j}\in Y$. Let us have a look at what happens on $\left(t_{h-1},t_{h}\right)$.

Punctual event $t_{h}$ can either be,

1. a removed leaf. The number of sampled lineages then goes from 1+x-y-w to x-y-w, and applying update (4.10) followed by Proposition 4.1 leads to
  M^(t,z)=λxψv+(w+1)+yrw+1(1-r)v+yR(tor-th,u(th-t,z))R(th-t,z)x-y-w∏tj∈XR(tj-th,u(th-t,z))∏tj∈WR(tj-th,u(th-t,z))-1∏tj∈Yu(tj-th,u(th-t,z))R(tj-th,u(th-t,z))-1=λxψv+(w+1)+yrw+1(1-r)v+yR(tor-t,z)R(th-t,z)R(th-t,z)x-y-w∏tj∈XR(tj-t,z)R(th-t,z)∏tj∈WR(th-t,z)R(tj-t,z)∏tj∈Yu(tj-t,z)R(th-t,z)R(tj-t,z)=λxψv+(w+1)+yrw+1(1-r)v+yR(tor-t,z)∏tj∈XR(tj-t,z)∏tj∈W∪{th}R(tj-t,z)-1∏tj∈Yu(tj-t,z)R(tj-t,z)-1.
  where the first to second equality is detailed in Appendix A, and the second to third comes after canceling out the $R(t_{h}-t,z)$.
2. a non-removed leaf. The number of sampled lineages then goes from 1+x-y-w to x-y-w, and applying update (4.11) followed by Proposition 4.1 leads to
  M^(t,z)=λxψv+w+(y+1)rw(1-r)v+(y+1)R(tor-t,z)R(th-t,z)R(th-t,z)x-y-wu(th-t,z)∏tj∈XR(tj-t,z)R(th-t,z)∏tj∈WR(th-t,z)R(tj-t,z)∏tj∈Yu(tj-t,z)R(th-t,z)R(tj-t,z)=λxψv+w+(y+1)rw(1-r)v+(y+1)R(tor-t,z)∏tj∈XR(tj-t,z)∏tj∈WR(tj-t,z)-1∏tj∈Y∪{th}u(tj-t,z)R(tj-t,z)-1.
3. a sampled ancestor. The number of sampled lineages then remains unchanged and equal to 1+x-y-w. Applying update (4.12) followed by Proposition 4.1 leads to
  M^(t,z)=λxψ(v+1)+w+yrw(1-r)(v+1)+yR(tor-t,z)R(th-t,z)R(th-t,z)1+x-y-w∏tj∈XR(tj-t,z)R(th-t,z)∏tj∈WR(th-t,z)R(tj-t,z)∏tj∈Yu(tj-t,z)R(th-t,z)R(tj-t,z)=λxψ(v+1)+w+yrw(1-r)(v+1)+yR(tor-t,z)∏tj∈XR(tj-t,z)∏tj∈WR(tj-t,z)-1∏tj∈Yu(tj-t,z)R(tj-t,z)-1.
4. a branching time. The number of sampled lineages then goes from 1+x-y-w to 2+x-y-w, and applying update (4.15) followed by Proposition 4.1 leads to
  M^(t,z)=λx+1ψv+w+yrw(1-r)v+yR(tor-t,z)R(th-t,z)R(th-t,z)2+x-y-w∏tj∈XR(tj-t,z)R(th-t,z)∏tj∈WR(th-t,z)R(tj-t,z)∏tj∈Yu(tj-t,z)R(th-t,z)R(tj-t,z)=λx+1ψv+w+yrw(1-r)v+yR(tor-t,z)∏tj∈X∪{th}R(tj-t,z)∏tj∈WR(tj-t,z)-1∏tj∈Yu(tj-t,z)R(tj-t,z)-1.

In all these cases, Proposition 4.3 is verified across epoch $\left(t_{h-1},t_{h}\right)$, which ends the proof.

## Appendix F Using a generating function to solve for $L_{t}$

### F.1 A slightly different strategy

Recall that $L_{t}$ verifies the following ODEs,

$$\begin{array}{cc} {\overset{˙}{L}}_{t}^{\left(i\right)}= & -\gamma (i+k)L_{t}^{\left(i\right)}+\lambda (2k+i)L_{t}^{\left(i+1\right)}+\mu {\mathrm{iL}}_{t}^{\left(i-1\right)}\\ L_{0}^{\left(i\right)}= & \rho_{0}^{k_{0}}{\left(1-\rho_{0}\right)}^{i}\text{.} \end{array}$$

If we introduce the corresponding generating function,

L^(t,z)=∑i=0∞ziLt(i)

then the initial condition on *L* translates into,

L^(0,z)=∑i=0∞(z(1-ρ))iρk0=ρk011-z(1-ρ),∀z∈±11-ρ.

The ODE translates into a PDE, but not as nicely as for $M_{t}$, see below,

∂tL^=∑i=0∞zi-γ(i+k)Lt(i)+λ(2k+i)Lt(i+1)+μiLt(i-1)=-γk∑i=0∞ziLt(i)-γ∑i=1∞iziLt(i)+λ∑i=1∞zi-1(2k+i-1)Lt(i)+μ∑i=0∞(i+1)zi+1Lt(i)=-γkL^-γz∂zL^+(2k-1)λ1z(L^-Lt(0))+λ∂zL^+μz2∂zL^+μzL^=-γk+(2k-1)λ1z+μzL^+(μz2-γz+λ)∂zL^-(2k-1)λ1zL^(t,0).

We are thus left with the following PDE problem,

(F.1) L^(0,z)=ρk01-z(1-ρ)-z∂tL^+(μz3-γz2+λz)∂zL^+(μz2-γkz+(2k-1)λ)L^-(2k-1)λL^(t,0)=0.

This remaining term with L^(t,0) complicates things a little bit. However, the initial condition on L^ provides us with a first candidate function to satisfy this PDE.

### F.2 Solution

We introduce below function *f*, and show that it satisfies the PDE problem (F.1).

$$f(t,z)≔\frac{p_{t}^{k}}{1-{\mathrm{zu}}_{t}}\text{.}$$

First, we observe that it satisfies the initial condition. We then need to check that it satisfies the PDE, and to do so we expand each of the four components of Eq. (F.1).

The first one gives us,

$$\begin{array}{cc} -z\partial_{t}f= & -z\frac{k{\overset{˙}{p}}_{t}p_{t}^{k-1}(1-{\mathrm{zu}}_{t})+z{\overset{˙}{u}}_{t}p_{t}^{k}}{{\left(1-{\mathrm{zu}}_{t}\right)}^{2}}\\ = & -z\frac{k(2\lambda u_{t}-\gamma )(1-{\mathrm{zu}}_{t})+(\lambda u_{t}^{2}-\gamma u_{t}+\mu )z}{{\left(1-{\mathrm{zu}}_{t}\right)}^{2}}p_{t}^{k}\\ = & \frac{\lambda (-2{\mathrm{kzu}}_{t}-(2k-1)z^{2}u_{t}^{2})+\gamma (\mathrm{kz}-(k-1)z^{2}u_{t})-\mu z^{2}}{{\left(1-{\mathrm{zu}}_{t}\right)}^{2}}p_{t}^{k}\text{.} \end{array}$$

We then turn to the second component,

$$(\mu z^{3}-\gamma z^{2}+\lambda z)\partial_{z}f=\frac{\lambda {\mathrm{zu}}_{t}-\gamma z^{2}u_{t}+\mu z^{3}u_{t}}{{\left(1-{\mathrm{zu}}_{t}\right)}^{2}}p_{t}^{k}\text{.}$$

And the third one,

$$\begin{array}{cc} (\mu z^{2}-\gamma \mathrm{kz}+(2k-1)\lambda )f= & \frac{\left(1-{\mathrm{zu}}_{t})(\mu z^{2}-\gamma \mathrm{kz}+(2k-1)\lambda \right)}{{\left(1-{\mathrm{zu}}_{t}\right)}^{2}}p_{t}^{k}\\ = & \frac{\lambda ((2k-1)-(2k-1){\mathrm{zu}}_{t})+\gamma (-\mathrm{kz}+{\mathrm{kz}}^{2}u_{t})+\mu (z^{2}-z^{3}u_{t})}{{\left(1-{\mathrm{zu}}_{t}\right)}^{2}}p_{t}^{k}\text{.} \end{array}$$

And the fourth and final one,

$$\begin{array}{cc} -(2k-1)\lambda f(t,0)= & \frac{-\lambda (2k-1){\left(1-{\mathrm{zu}}_{t}\right)}^{2}}{{\left(1-{\mathrm{zu}}_{t}\right)}^{2}}p_{t}^{k}\\ = & \frac{\lambda (-(2k-1)z^{2}u_{t}^{2}+2(2k-1){\mathrm{zu}}_{t}-(2k-1))}{{\left(1-{\mathrm{zu}}_{t}\right)}^{2}}p_{t}^{k}\text{.} \end{array}$$

Putting everything together, we can now check that indeed,

$$-z\partial_{t}f+(\mu z^{3}-\gamma z^{2}+\lambda z)\partial_{z}f+(\mu z^{2}-\gamma \mathrm{kz}+(2k-1)\lambda )f-(2k-1)f(t,0)=0\text{.}$$

While the branching and ψ-sampling with removal updates do not change anything to this solution, all the others do. Further work is thus needed to look for other solutions to this same PDE with different initial conditions.
